# Diversity and geographical distribution of potential carbon monoxide oxidizers using molybdenum-containing enzymes in the ocean

**DOI:** 10.1128/msphere.00062-26

**Published:** 2026-04-23

**Authors:** Yoshinari Imaura, Keigo Yamamoto, Ryoma Kamikawa, Takashi Yoshida

**Affiliations:** 1 Graduate School of Agriculture, Kyoto University, Sakyo-ku, Kyoto, Japan; 2 Graduate School of Agricultural and Life Sciences, The University of Tokyo, Bunkyo-ku, Tokyo, Japan; 3 Research Institute of Environment, Agriculture and Fisheries, Osaka Prefecture 52741, Osaka, Japan; University of Wisconsin-Madison, Madison, Wisconsin, USA

**Keywords:** carbon monoxide, ocean, atmosphere, biogeography, microbial ecology

## Abstract

Carbon monoxide (CO) evolves from photochemical or thermal reactions in the ocean. It is estimated that 90% of oceanic CO is consumed by CO-oxidizing prokaryotes, which convert CO to carbon dioxide using molybdenum-containing CO dehydrogenase (Mo-CODH). Investigation of form I *cox*, which encodes Mo-CODH, has revealed that oceanic prokaryotes with form I *cox* (potential *cox*-containing CO oxidizers: p*cox*-CO oxidizers) belong to eight phyla and occupy 10%–20% of prokaryotes. However, previous studies may have overestimated their diversity due to the use of less stringent criteria, and their ecology remains poorly understood. In this study, we characterized p*cox*-CO oxidizers by identifying form I *cox* from prokaryotic genomes reconstructed from the ocean. We used criteria that considered the phylogeny of Cox, their active site motifs, and the operon structure of *cox*. As a result, 233 species from nine phyla, which included 207 species unknown to be p*cox*-CO oxidizers, were found. We investigated the biogeography of each species and found 34 species that dominate in particular oceanic regions. Among the 34 species, 11 co-occurred with either of 20 prokaryotic species, and the co-occurring partners varied among species. No functional genes except those related to CO oxidation were shared between the 11 species, which implied the absence of common molecular basis that underlies ecological interaction between p*cox*-CO oxidizers and other prokaryotes. Finally, we performed absolute quantification of four species of p*cox*-CO oxidizers that were predicted to be dominant in Osaka Bay, Japan. It showed that p*cox*-CO oxidizers occupied over 8.49% of the bacterial community.

**IMPORTANCE:**

The ocean is a source of carbon monoxide (CO), an indirect greenhouse gas that supports the accumulation of methane and the production of a precursor of tropospheric ozone. The primary sink of CO in the ocean is prokaryotic CO oxidizers which possess molybdenum-containing CO dehydrogenase (Mo-CODH). Understanding CO flux therefore requires ecological characterization of prokaryotes carrying *cox,* which encode Mo-CODH. We provide a comprehensive, well-curated catalog of such prokaryotes (potential *cox*-containing CO oxidizers: p*cox*-CO oxidizers) in the ocean that not only revealed their diversity but also enabled species-specific ecological assessments. Co-occurrence analyses and genomic analysis of p*cox*-CO oxidizers uncovered substantial variation in their co-occurring prokaryotic partners and functional gene repertoires. The lack of shared co-occurrence and conserved genes suggests that CO oxidation via Mo-CODH does not mediate ecological interactions. These findings provide a foundation for future studies of p*cox*-CO oxidizers and offer new insight into ecological roles of CO oxidizers.

## INTRODUCTION

Carbon monoxide (CO) is an atmospheric trace gas present at tens to hundreds of ppb (1–5). It acts as an indirect greenhouse gas by consuming hydroxyl radicals that remove methane (6) and by providing a precursor to tropospheric ozone (7). CO is produced in the ocean through photochemical or thermal reactions (8–11) and emitted into the atmosphere (12). Oceanic CO emission (estimated to be 4 Tg C/year) (12) is minor in the global budget (estimated to be 2,600 Tg C/year), which is largely accounted for by anthropogenic emission, biomass burning, and hydrocarbon oxidation (13). However, oceanic CO may have a critical impact on remote oceans, where other CO sources are scarce.

It is estimated that 90% of oceanic CO is removed before emission to the air by prokaryotes called CO oxidizers that convert CO to carbon dioxide using molybdenum-containing CO dehydrogenase (Mo-CODH) (12, 14). There is also a distinct group of CO oxidizers, which lack Mo-CODH but possess structurally different nickel-containing CO dehydrogenase (Ni-CODH). CO oxidizers with Ni-CODH oxidize elevated levels of CO (20%–100%) (15, 16). Mo-CODH-mediated CO oxidation is coupled with the reduction of oxygen, nitrate, or perchlorate (17–19). The large subunit of Mo-CODH (CoxL), a xanthine oxidase family protein (20), is classified into two groups (form I and form II) (17). They are distinguished by active site motifs (AYXCSFR vs AYRGAGR) and by the order of genes encoding large, medium, and small subunits (*coxL*, *coxM*, and *coxS*, respectively) in the operon (*coxMSL* vs *coxSLM*) (17). The role of form II Cox remains unclear because isolates possessing only form II *cox* often show no detectable CO consumption (21), whereas form I Cox is considered responsible for CO oxidation (22–24). CO oxidizers and prokaryotes with *cox* (potential *cox*-containing CO oxidizers: p*cox*-CO oxidizers) have been studied using culture-dependent and culture-independent surveys (22, 25, 26). To date, 13 species from six genera (*Stappia*, *Ruegeria*, *Roseovarius*, *Dinoroseobacter*, *Roseobacter*, and *Planktomarina*) in the phylum *Pseudomonadota,* isolated from the ocean, are characterized as CO oxidizers (21, 27–29). Additional p*cox*-CO oxidizers have been reported from at least eight phyla (*Acidobacteriota*, *Actinomycetota*, *Bacteroidota*, *Chloroflexota*, *Gemmatimonadota*, *Myxococcota*, *Pseudomonadota*, and SAR324) (22, 30, 31) by analyzing metagenome-assembled genomes (MAGs), single-amplified genomes (SAGs), and isolate genomes from marine environments. Read mapping analyses have suggested that they constitute 10%–20% of marine prokaryotes on average (22, 32, 33) and are enriched in coastal areas during spring (34) and in the deep ocean (22). However, their diversity and ecology are poorly understood. The relative abundance of p*cox*-CO oxidizers might be overestimated since previous surveys relied on homology search of *coxL* and annotations based on Kyoto Encyclopedia of Genes and Genomes orthology (35) or Clusters of Orthologous Groups (COG) (36), which can also detect form II CoxL. Conservation of active sites and operon structures has likewise not been fully considered. Consequently, the species-level ecological features of p*cox*-CO oxidizers remain unclear.

In this study, we addressed these issues by constructing a catalog of p*cox*-CO oxidizers following stringent criteria incorporating phylogeny and active site motifs of CoxL as well as operon structures of *cox*. Using this catalog, we characterized the biogeography of p*cox*-CO oxidizers. We identified dominant species and performed co-occurrence analyses to infer factors shaping their biogeography. We further analyzed the pangenome of p*cox*-CO oxidizers to characterize conserved functions potentially underlying ecological interactions with other prokaryotes. Finally, we designed qPCR primers that enabled species-specific quantification of four p*cox*-CO oxidizer species predicted to dominate in Osaka Bay, Japan.

## RESULTS

### Detection of p*cox*-CO oxidizers

To accurately detect p*cox*-CO oxidizers, we searched for genes encoding CoxL-like proteins using homology search, followed by filtering based on active site motifs, phylogeny, and *coxMSL* operon structures (Fig. S1). These processes were more stringent than those of previous studies, which did not necessarily consider active site motifs or operon structures (8, 25).

We investigated previously reported CoxL active site motifs (23). Among 707 form I CoxLs, we identified six motifs—AYACSFR, AYRCSFR, SYRCSFR, AYRCSLR, AYSCSFR, and SYACSFR. Since these were homologous to previously proposed motif (AYXCSFR) (17), we regarded them as genuine motifs. Among the six, AYRCSLR may be less functional due to a substitution in active site CSFR residues (37). Nevertheless, inclusion of AYRCSLR had minimal impact (additional analyses and results in Supplemental material).

We retrieved 1,328 prokaryotic genomes (hereafter called the non-OceanDNA MAGs) that have been reported to possess *cox* in 59 studies (Table S1) to examine our screening process. Homology search identified 9,462 genes encoding CoxL-like proteins in 1,063 genomes. Only 329 of 9,462 genes from 289 genomes possessed active site motifs and formed a monophyletic group with form I CoxL of *Afipia carboxidovorans* (AEI08106.1), which has been biochemically shown to oxidize CO (38) (Fig. S2). Accordingly, we identified them as form I CoxL. Of these, 254 genes in 235 genomes were located downstream of *coxM* and *coxS*, forming *coxMSL* gene operons (Fig. S1; Table S2). We identified these genomes as p*cox*-CO oxidizers. Since *coxMSL* gene operons might be split into several contigs, the number of genomes might be underestimated. Nevertheless, we detected genomes of all five isolates characterized as CO oxidizers whose genomes were available (21, 27).

We applied the screening to a catalog of MAGs constructed from metagenome data collected from various sites in the global ocean (the OceanDNA MAGs, *n* = 52,325) (39). We identified 119,560 CoxL-like proteins in 26,690 genomes. Of these, 1,924 proteins in 1,870 genomes were identified as form I CoxL. Of the 1,924, 1,283 in 1,279 MAGs were encoded in *coxMSL* gene operons (Fig. S1; Table S2). In addition to 1,279 MAGs, two MAGs which were classified in phyla *Marinisomatota* and *Pseudomonadota* had notable *coxL*-like genes harboring active site-like motifs (AYRCSSR and AYRCSCR, respectively), and the *coxL*-like gene of the latter MAG was located within a *coxMSL*-like operon. Further analyses suggested that these genes arose from point mutations in form I *coxL* (additional analyses and results, Fig. S3, and Table S3 in Supplemental material). However, these were excluded because such motifs had not been reported.

**Fig 1:**
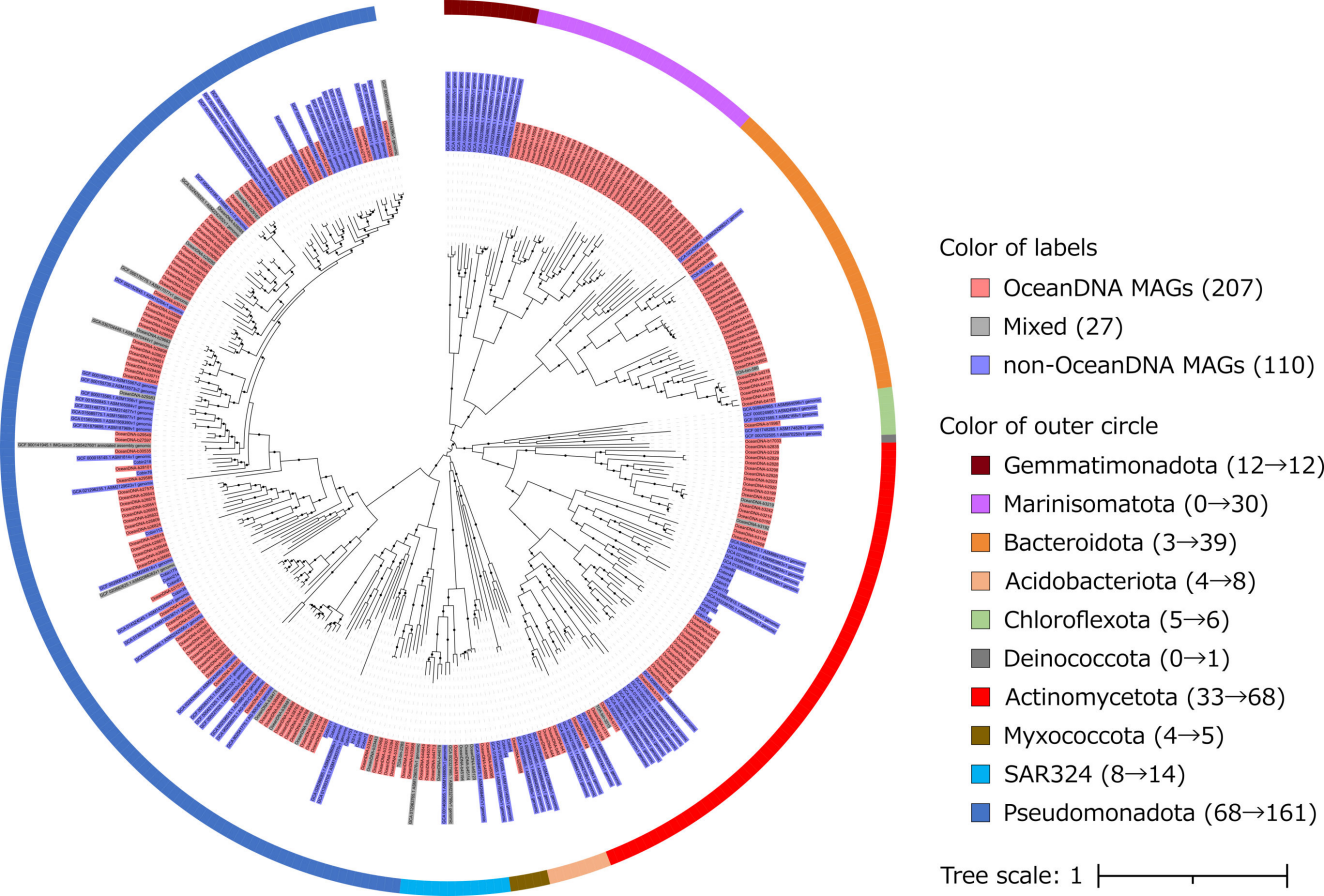
Phylogenetic tree of representative genomes of species clusters from which p*cox*-CO oxidizers were identified. Branches with bootstrap values of ≥90 are highlighted by black dots. Branch lengths represent the number of substitutions per site. The OTU label color represents the source of the genomes, which belong to the same cluster as representative genomes. Species clusters composed only of the OceanDNA MAGs (labeled “OceanDNA MAGs”), composed of both the OceanDNA and non-OceanDNA MAGs (labeled “mixed”), and composed of only the non-OceanDNA MAGs (labeled “non-OceanDNA MAGs”) are highlighted in red, gray, and blue, respectively. In parentheses are the number of species clusters in each category. Colors that make up the circle represent the phyla to which each representative genome belongs. The number to the left of the phyla name represents how much the exploration against the OceanDNA MAGs contributes to the expansion of the diversity of p*cox*-CO oxidizers. The number of species clusters before the exploration against the OceanDNA MAGs and after the exploration is indicated by numbers to the right and left of the arrow, respectively. Two archaeal species clusters composed solely of the non-OceanDNA MAGs are not included in the phylogenetic tree.

The p*cox*-CO oxidizer genomes were grouped into 346 species-level genome clusters (species clusters) under the average nucleotide identity (ANI) >95%, which represents the species boundary (40). The species clusters belonged to *Acidobacteriota*, *Actinomycetota*, *Bacteroidota*, *Chloroflexota*, *Deinococcota*, *Gemmatimonadota*, *Marinisomatota*, *Myxococcota*, *Pseudomonadota*, SAR324, *Thermoproteota*, and an unclassified archaeal phylum. Among the 346, species clusters, including the non-OceanDNA MAGs, represent previously known p*cox*-CO oxidizers, whereas species clusters exclusively comprised of the OceanDNA MAGs represent newly identified p*cox*-CO oxidizers. Of the 346 clusters, 207 contained only OceanDNA MAGs, 112 only non-OceanDNA MAGs, and 27 both (Fig. 1). We thus expanded the catalog of p*cox*-CO oxidizers by adding 207 species. The 207 species clusters spanned 12 classes, 26 orders, 39 families, and 115 genera in nine phyla (*Acidobacteriota*, *Actinomycetota*, *Bacteroidota*, *Chloroflexota*, *Deinococcota*, *Marinisomatota*, *Myxococcota*, *Pseudomonadota*, and SAR324). Among the 234 species clusters including the OceanDNA MAGs, 114 included both *cox*-containing and *cox*-lacking genomes (Table S4), suggesting that certain populations in these species clusters might have potential to oxidize CO. We note that two species clusters in the family *Burkholderiaceae*, the phylum *Pseudomonadota,* were originally classified as one species in the OceanDNA MAGs (39). We merged them into one species cluster (1206_1) to maintain consistency.

### Dominant p*cox*-CO oxidizers exhibited species-level niche separation

To characterize ecological features of p*cox*-CO oxidizers derived from the OceanDNA MAGs, we investigated the biogeography of their representative genomes. We retrieved metagenome data of 1,134 samples (Table S5) collected using filters of 0.1 μm–0.8 μm pore size from distinct depths and times at 354 sites via previous studies (Table 1). Metagenome reads were mapped onto 8,466 representative genomes of species clusters including 233 genomes of p*cox*-CO oxidizers. We calculated the reads per kilobase per million (RPKM) values of 14 single-copy marker genes (Table S6) used in a previous literature (22) and calculated the relative abundances of each genome based on the average RPKM values of the 14 genes. This value served as a proxy for the relative abundance of the whole community of each species cluster. The relative abundance of p*cox*-CO oxidizers was calculated using RPKM values of *coxMSL* gene operons. Relative abundance was obtained for 8,427 genomes, including 231 p*cox*-CO oxidizer genomes, which contained any of the 14 genes.

**TABLE 1:** Expedition projects and time course and/or depth-resolved sampling through which metagenome data of the 1,134 samples were obtained

Expedition projects and time course or depth-resolved sampling	Reference
Tara Ocean	(41)
Malaspina	(32)
GEOTRACES	(42)
Medea	(43)
Expeditions in the Baltic Sea	(44)
Expeditions in the Red Sea	(45)
Time course and depth-resolved sampling in Saanich Inlet, Canada	(46)
Time course and depth-resolved sampling in Monterey Bay, USA	(47)
Time course sampling in SOLA Station, France	(48)
Time course and depth-resolved sampling in Hawaii	(49, 50)
Depth-resolved sampling in Eastern Tropical North Pacific (ETNP) off the coast of Mexico	(51)

**Fig 2:**
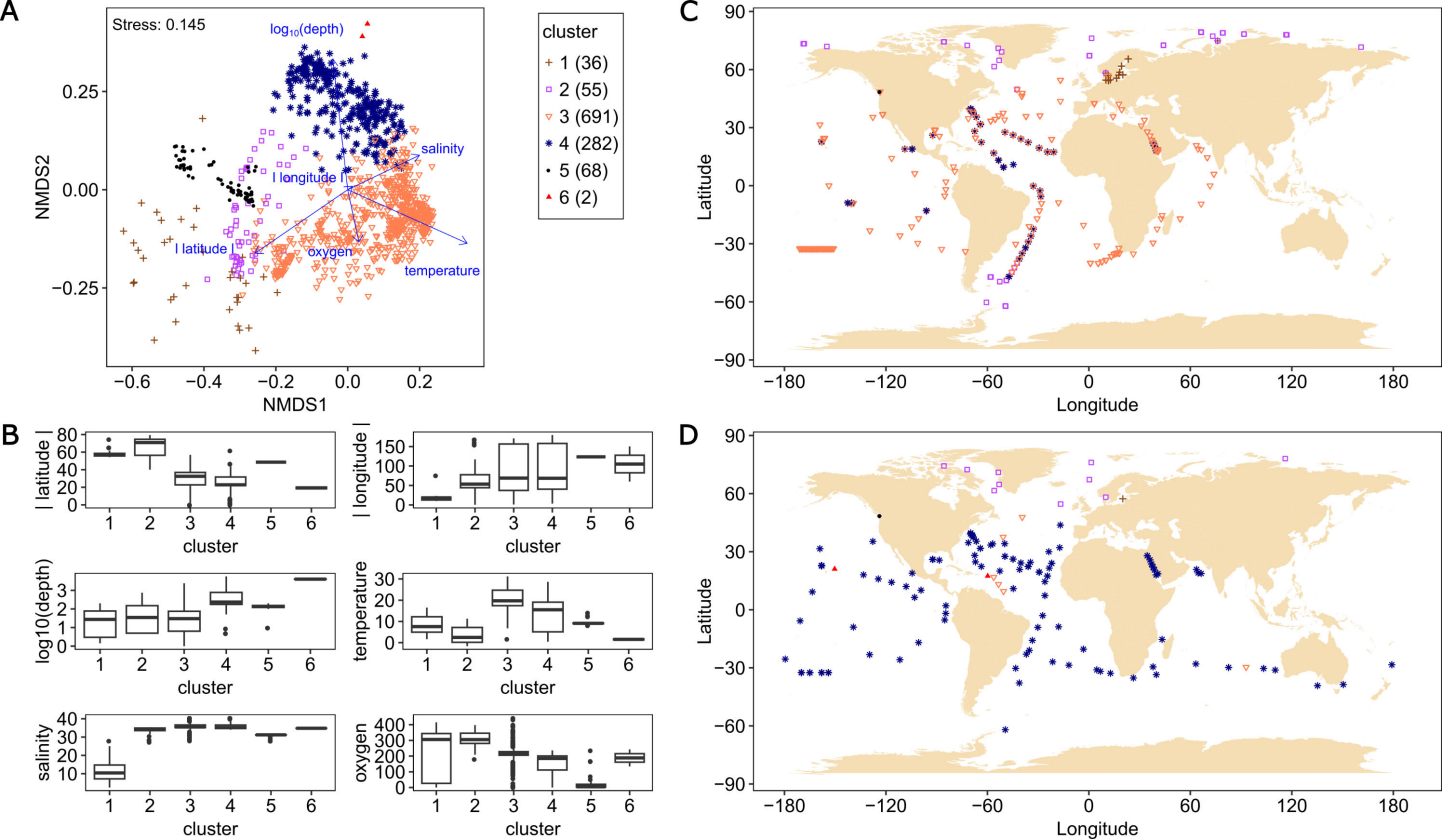
Characteristics of geographic locations and environmental conditions of samples from which metagenome data of each metagenomic cluster are derived. (**A**) Nonmetric multidimensional scaling plots of the prokaryotic community of 1,134 metagenome data calculated using the Bray-Curtis distance matrix. Arrows indicate the vectors that correlate with six environmental parameters (absolute value of latitude, absolute value of longitude, common logarithm of depth, temperature, oxygen concentration, and salinity). The colors and shapes of plots correspond to the metagenomic clusters. The number of metagenomes in each metagenomic cluster is shown in parentheses in the legend. (**B**) Box and whisker plots representing the distribution of environmental parameters of samples from which metagenomes of each metagenomic cluster are derived. (**C**) Geographic locations of sampling sites from the surface ocean (depth < 200 m). (**D**) Geographic locations of sampling sites from the deep ocean (depth ≥ 200 m). The colors and shapes of plots in panels **C** and **D** correspond to the metagenomic clusters, which are the same as in panel **A**.

**Fig 3:**
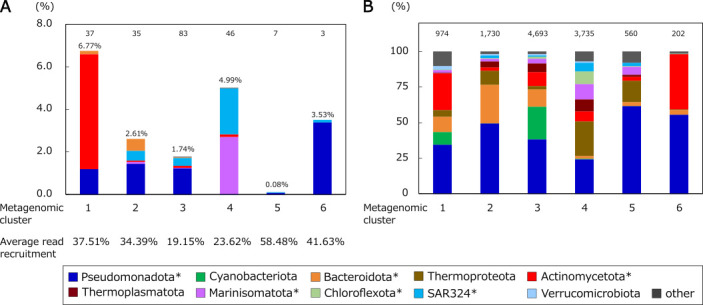
Phylum-level community composition of (**A**) p*cox*-CO oxidizers and (**B**) total prokaryotes. The average relative abundance of each species cluster is summed to calculate the relative abundance of each phylum. Colors of the bars represent the phylum. Asterisks indicate the phyla in which p*cox*-CO oxidizers were found. The numbers at the top of panels **A and B** represent the numbers of observed species clusters with p*cox*-CO oxidizers and numbers of all species clusters, respectively. In panel **A**, the cumulative relative abundance of p*cox*-CO oxidizers is shown above each bar. Numbers below panel **A** indicate the average read recruitment (the proportion of metagenome reads mapped to reference genomes relative to total reads) for each metagenomic cluster.

A Mantel test was carried out to reveal correlation between prokaryotic community composition, composition of p*cox*-CO oxidizers, and environmental parameters (depth, latitude, longitude, temperature, salinity, and oxygen concentration). Both compositions exhibited a positive relationship (*P*-value ≤0.001, Mantel’s *r* values of 0.30–0.58) for all environmental parameters except for longitude (Fig. S4). This suggests a determining role for these parameters, as observed in previous studies of prokaryotic communities (52–55). Spearman’s correlation test revealed that all environmental parameters were significantly correlated with each other (*P* < 0.001), except for depth and salinity (Fig. S4). There were particularly strong positive correlations between temperature and salinity and between oxygen concentration and latitude (correlation coefficients > 0.55). There were high negative correlations between temperature and latitude and between oxygen concentration and depth (correlation coefficients < −0.51) (Fig. S4). It suggests that these parameters do not always shape communities independently. From these results, we assumed that metagenome samples with similar prokaryotic community compositions originated from similar environments and would harbor similar p*cox*-CO oxidizer compositions. Thus, we clustered the 1,134 metagenome data sets based on prokaryotic community compositions and compared the composition of p*cox*-CO oxidizers in each metagenomic cluster. When the metagenome data sets were clustered into 5 to 10, 6 were adopted since the resultant metagenomic clusters were the most stable when randomly selected subsets of data were repeatedly clustered (Fig. S5). The six metagenomic clusters were characterized by environmental conditions and locations of sampling sites: brackish region of the Baltic Sea (cluster 1), polar oceans (cluster 2), surface layer of temperate/tropical oceans (cluster 3), mesopelagic, bathypelagic, or hadalpelagic layers (cluster 4), anoxic water mass of Saanich Inlet (cluster 5), and bathypelagic layer of Pacific/Atlantic oceans (cluster 6) (Fig. 2). The average read recruitment (proportion of mapped reads to total reads) differed among metagenomic clusters: highest in cluster 5 (58.5%), followed by clusters 6, 1, and 2 (34.4%–41.6%), and low in clusters 3 and 4 (19.1% and 23.6%, respectively) (Fig. S6). Average relative abundance of p*cox*-CO oxidizers also varied; highest in cluster 1 (6.77%), lowest in cluster 5 (0.08%), and ranged from 1.74% to 4.99% in other clusters (Fig. 3A). The low relative abundance in cluster 5 (Saanich Inlet) despite the high read recruitment (Fig. S6) suggests that p*cox*-CO oxidizers identified here are minor in oxygen-depleted waters.

**Fig 4:**
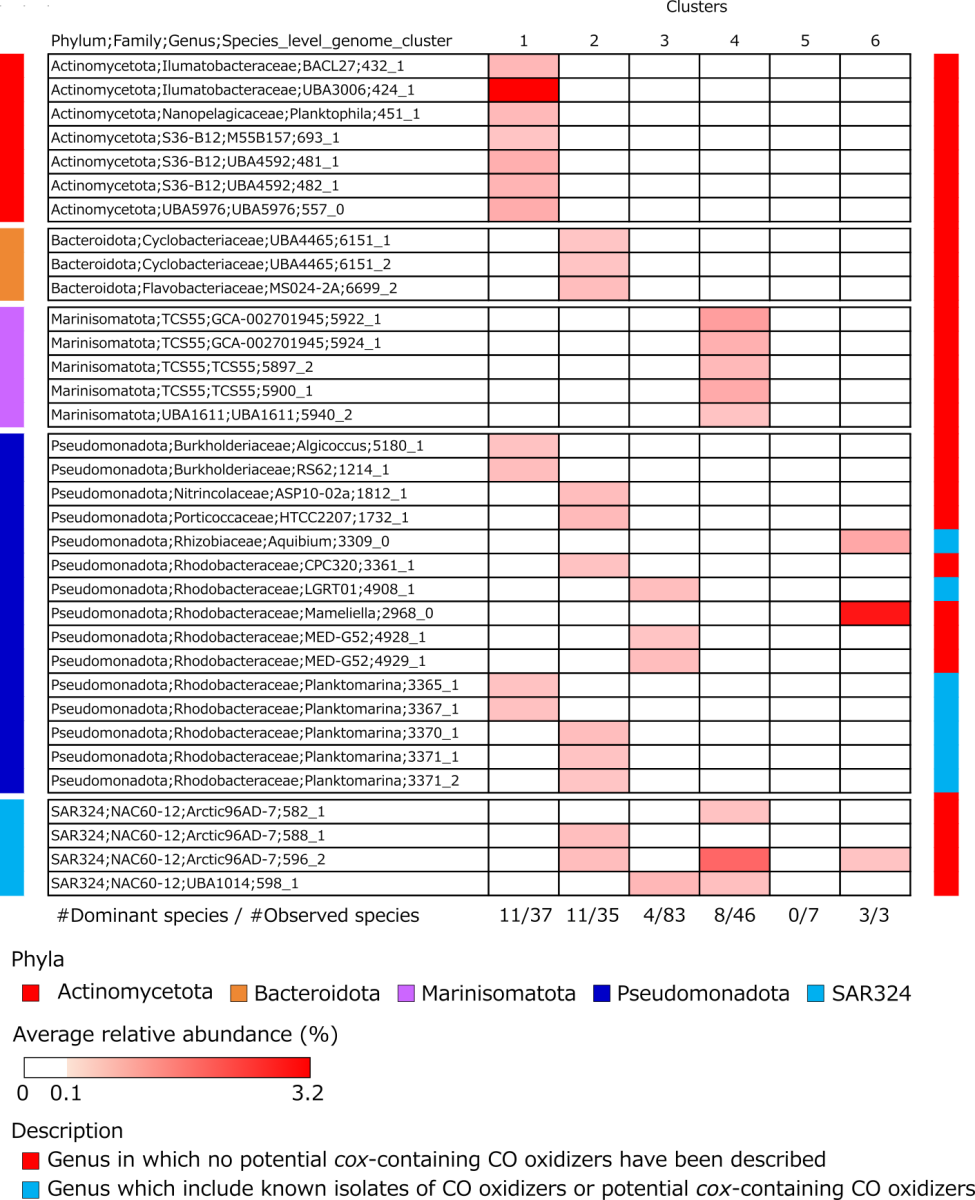
Average relative abundance of dominant species clusters with p*cox*-CO oxidizers in six metagenomic clusters. The color gradient of the heatmap represents the average relative abundance of representative genomes of p*cox*-CO oxidizers in species clusters in each metagenomic cluster. Colors to the left of the heatmap indicate phyla, and colors on the right of the heatmap indicate whether *cox*-containing CO oxidizers or p*cox*-CO oxidizers had been identified in the same genera as corresponding species clusters. Numbers below the heatmap indicate the ratio of the number of dominant (average relative abundance >0.1%) species clusters to that of observed species clusters, including p*cox*-CO oxidizer genomes.

As predicted by the Mantel test, community composition of p*cox*-CO oxidizers differed among metagenomic clusters. *Actinomycetota* dominated in cluster 1 (occupied 80%), while *Marinisomatota* and SAR324 dominated in cluster 4 (occupied 97% in total) (Fig. 3A). *Pseudomonadota* dominated in clusters 2, 3, and 6 (occupied 55%–96%) (Fig. 3A). These compositions differed from those of total prokaryotic communities. For example, *Pseudomonadota* occupied 17% of p*cox*-CO oxidizers but 35% of prokaryotic community in clusters 1 (Fig. 3). This implies habitat-specific enrichment of p*cox*-CO oxidizers, consistent with a previous observation (34). Thus, we characterized the distribution of dominant species clusters of p*cox*-CO oxidizers, which are defined as those with ≥0.1% average relative abundance in any metagenomic cluster. We found 34 dominant species clusters across 23 genera and five phyla (*Actinomycetota*, *Bacteroidota*, *Marinisomatota*, *Pseudomonadota*, and SAR324) (Fig. 4). Of these, seven belonged to three genera in the phylum *Pseudomonadota* (*Aquibium*, LGRT01, and *Planktomarina*), from which *cox*-containing CO oxidizers and p*cox*-CO oxidizers were isolated (27, 56, 57) (Fig. 4). Although the “genus LGRT01” is not effectively published, we treat it and other clades without published names as genera following classification by GTDB-Tk v.2.4.0 (58). Dominant species clusters exhibited distinct distribution patterns. For example, in the genus *Planktomarina*, species clusters 3370_1, 3371_1, and 3371_2 were enriched in cluster 2 (polar oceans), whereas species clusters 3365_1 and 3367_1 were enriched in cluster 1 (Baltic Sea) (Fig. 4). These results indicated species-level niche separation among p*cox*-CO oxidizers. Such niche separation is also observed in other functional groups such as phytoplankton (59) and diazotrophs (60).

### Dominant p*cox*-CO oxidizers co-occurred with other prokaryotes in a species-specific manner

**Fig 5:**
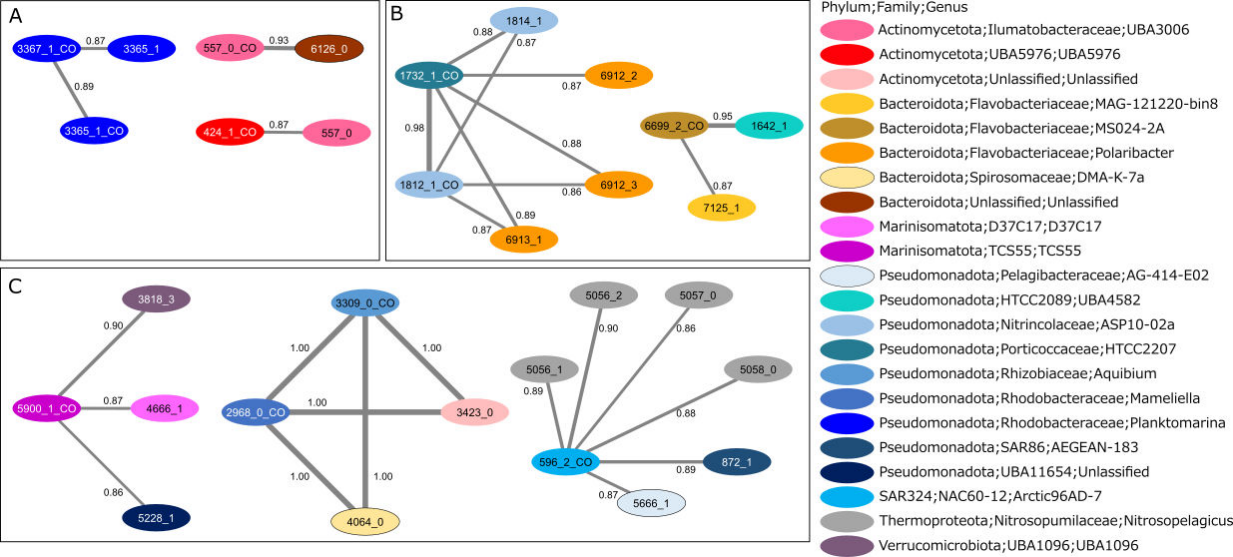
Co-occurrence network of p*cox*-CO oxidizers and other prokaryotes of which relative abundance was the highest in (**A**) metagenomic cluster 1, (**B**) metagenomic cluster 2, and (**C**) metagenomic clusters 4 and 6. Each node represents p*cox*-CO oxidizers (nodes with “_CO”) or the whole community of species clusters (nodes without “_CO”). The nodes are colored by genera. Significant positive Spearman’s correlations (ρ ≥0.85, false discovery rate-adjusted *P* < 0.01) are graphed as edges. The thickness of the edges corresponds to the ρ value, which is displayed on the edges.

We performed a co-occurrence network analysis to predict biotic and abiotic factors shaping species-specific distributions. To extract factors uniquely affecting abundance of p*cox*-CO oxidizers, we retrieved correlations that included p*cox*-CO oxidizers but did not include the whole community of the species clusters. The resultant network contained 31 nodes (p*cox*-CO oxidizers or the whole communities of species clusters) and 28 edges (correlation with |*r*| ≥ 0.85 and *P* < 0.01), forming eight modules. Neither correlations between environmental parameters nor negative correlations were observed. Three of the eight modules comprised species clusters with the highest relative abundance in metagenomic cluster 1 (Baltic Sea). The p*cox*-CO oxidizers of 3367_1 in the genus *Planktomarina* co-occurred with species cluster 3365_1, another member of *Planktomarina* (Fig. 5A). p*cox*-CO oxidizers in two additional species clusters in uncultured genera of the phylum *Actinomycetota* (424_1 and 557_0) co-occurred with species cluster 557_0 and with a species cluster in an unclassified lineage of the phylum *Bacteroidota*, respectively (Fig. 5A). Two of the eight modules comprised species clusters with the highest relative abundance in metagenomic cluster 2 (polar oceans). The p*cox*-CO oxidizers in species clusters 1732_1 and 1812_1, which belonged to uncultured genera HTCC2207 and ASP10-02a, respectively, in the phylum *Pseudomonadota* co-occurred with each other. They also co-occurred with another species cluster in the genus ASP10-02a, and with three species clusters in the genus *Polaribacter* (phylum *Bacteroidota*) (Fig. 5B). The p*cox*-CO oxidizers in species cluster 6699_2 (a member of the uncultured genus MS024-2A, phylum *Bacteroidota*) co-occurred with two species clusters 1642_1 and 7125_1, which belonged to uncultured genera HTCC2089 in the phylum *Pseudomonadota* and MAG-121220-bin8 in the phylum *Bacteroidota*, respectively (Fig. 5B). The remaining three modules comprised species clusters with the highest relative abundance in metagenomic clusters 4 or 6 (mesopelagic to hadalpelagic layers). The p*cox*-CO oxidizers in species cluster 5900_1 (a member of the uncultured genus TCS55 in the phylum *Marinisomatota*) co-occurred with three species clusters (3818_3, 4666_1, and 5228_1), which belonged to uncultured lineages (the genus UBA1096 in the phylum *Verrucomicrobiota*, the genus D37C17 in the phylum *Marinisomatota*, and the family UBA11654 in the phylum *Pseudomonadota*, respectively) (Fig. 5C). The p*cox*-CO oxidizers in species clusters 3309_1 and 2968_1 (members of genera *Aquibium* and *Mameliella*, respectively, in the phylum *Pseudomonadota*) co-occurred with two species clusters (3423_0 and 4064_0), which belonged to uncultured lineages (an unclassified genus in the phylum *Actinomycetota* and the genus DMA-K-7a in the phylum *Bacteroidota*, respectively) (Fig. 5C). The p*cox*-CO oxidizers in species cluster 596_2 (a member of the uncultured genus Arctic96AD-7 in the phylum SAR324) co-occurred with six species clusters belonging to the genus *Nitrosopelagicus* (MGI archaea [61]) in the phylum *Thermoproteota*, the uncultured genus AG-414-E02 in the SAR11 clade, and the uncultured genus AEGEAN-183 in the SAR86 clade (Fig. 5C).

### No functional genes were conserved among p*cox*-CO oxidizers across species clusters

To investigate genetic traits underlying co-occurrence, we analyzed the pangenome of ten species clusters (1732_1, 1812_1, 2968_0, 3309_0, 3367_1, 424_1, 557_1, 5900_1, 596_2, 6699_2) in which p*cox*-CO oxidizers co-occurred with other prokaryotes. We retrieved genes that were located outside the *coxMSL* gene operon, present in MAGs of p*cox*-CO oxidizers, but not found in *coxL*-lacking MAGs. Seven species clusters, except 2968_0, 3309_0, and 557_1, contained both p*cox*-CO oxidizer MAGs and *coxL*-lacking MAGs. In each of the seven, 7–144 (in total 187) COGs were identified exclusively from p*cox*-CO oxidizer MAGs (Table S7). Of the 187 COGs, 7 (COG3427, COG3552, COG2068, COG1231, COG0840, COG3344, and COG3378) appeared across multiple species clusters (Table 2). However, six COGs, except for COG2068 (MocA), were not consistently detected in p*cox*-CO oxidizer MAGs across the seven species clusters (Table 2). As MocA participates in biosynthesis of a metal cofactor in molybdoenzymes including Cox (62), conservation of COG2068 suggested a functional association with CO oxidation. These findings indicated the absence of conserved genes which provide a common basis for ecological interaction between p*cox*-CO oxidizers and other prokaryotes.

**TABLE 2:** COGs which were identified in the pangenome of p*cox*-CO oxidizers (*coxMSL*+) but not identified in the pangenome of *coxL*-lacking genomes (*coxL*−) in multiple species clusters and the numbers of genomes which possessed each of the COG in each species cluster^*a*^

COG	Description	1732_1		1812_1		3367_1		424_1		5900_1		596_2		6699_2	
		*cox* *MSL*+	*coxL*−	*cox* *MSL*+	*coxL*−	*cox* *MSL*+	*coxL*−	*cox* *MSL*+	*coxL*−	*cox* *MSL*+	*coxL*−	*cox* *MSL*+	*coxL*−	*cox* *MSL*+	*coxL*−
#Genome		1	45	4	92	10	60	35	5	2	86	20	99	3	8
COG 3427	Carbon monoxide dehydrogenase subunit CoxG	1 (1.00)	0 (0.00)	4 (1.00)	0 (0.00)	10 (1.00)	60 (1.00)	35 (1.00)	5 (1.00)	0 (0.00)	0 (0.00)	15 (0.75)	80 (0.81)	3 (1.00)	0 (0.00)
COG 3552	Uncharacterized protein CoxE	1 (1.00)	0 (0.00)	4 (1.00)	1 (0.01)	10 (1.00)	60 (1.00)	35 (1.00)	5 (1.00)	0 (0.00)	0 (0.00)	18 (0.90)	60 (0.61)	3 (1.00)	0 (0.00)
COG 2068	CTP:molybdo-pterin cytidyl-transferase MocA	1 (1.00)	34 (0.76)	2 (0.50)	0 (0.00)	9 (0.90)	56 (0.93)	34 (0.97)	4 (0.80)	1 (0.50)	40 (0.47)	16 (0.80)	75 (0.76)	3 (1.00)	0 (0.00)
COG 1231	Monoamine oxidase MaoA	0 (0.00)	2 (0.04)	0 (0.00)	0 (0.00)	2 (0.20)	0 (0.00)	1 (0.03)	00 (0.00)	2 (1.00)	83 (0.97)	0 (0.00)	0 (0.00)	0 (0.00)	0 (0.00)
COG 0840	Methyl-accepting chemotaxis protein (MCP)	0 (0.00)	1 (0.02)	4 (1.00)	90 (0.98)	1 (0.10)	0 (0.00)	1 (0.03)	0 (0.00)	0 (0.00)	1 (0.01)	3 (0.15)	7 (0.07)	0 (0.00)	0 (0.00)
COG 3344	Retron-type reverse transcriptase YkfC	0 (0.00)	0 (0.00)	1 (0.25)	0 (0.00)	0 (0.00)	10 (0.16)	1 (0.02)	0 (0.00)	0 (0.00)	0 (0.00)	5 (0.25)	6 (0.06)	0 (0.00)	0 (0.00)
COG 3378	DNA primase, phage- or plasmid-associated	0 (0.00)	0 (0.00)	0 (0.00)	5 (0.05)	0 (0.00)	9 (0.15)	2 (0.06)	0 (0.00)	0 (0.00)	1 (0.01)	2 (0.1)	7 (0.07)	2 (0.67)	0 (0.00)

^*a*^ Values in the parentheses are proportion of genomes in which each of the COG was detected among all genomes in each species cluster.

### Species-specific quantification revealed dominance of p*cox*-CO oxidizers in Osaka Bay

The abovementioned analyses on the relative abundance revealed dominant p*cox*-CO oxidizers, which might be ecologically important. However, relative abundance does not necessarily reflect absolute abundance (63). Since existing quantitative analyses of p*cox*-CO oxidizers have focused on total *coxL* copy density (64), absolute abundance remains unresolved at the species level. We therefore performed absolute quantification using species-specific real-time PCR primers targeting *coxL*. Surface seawater was sampled on 22 June 2022, from a 5 m depth in Osaka Bay, Japan—a site not included in the data set used for abovementioned analyses. Because the water temperature (21.58°C) fell within the range of temperatures of samples in metagenomic cluster 3 (1.82°C–31.2°C), we targeted four dominant species clusters from this metagenomic cluster: 4908_1 (a member of the genus LGRT01), 4928_1 (a member of the uncultured genus MED-G52 in the phylum *Pseudomonadota*), 4929_1 (another member of the genus MED-G52), and 598_1 (a member of the uncultured genus UBA1014 in the phylum SAR324). We constructed species-specific real-time primers (Table 3). We note that *coxL* of 4908_1 and two other species clusters, 4908_2 and 4908_3 (uncultured members of the genus LGRT01), were not distinguished due to their high sequence similarity and were detected using the same primers.

**TABLE 3:** Primers used in this study^*a*^

Primer	Sequence (5′ to 3′)	References
LGRT01_4908_1 to 3_coxL_2_f	GCAGATCCYACCAAATTTCC	This study
LGRT01_4908_1 to 3_coxL_2_r	ACGCGRAAGGAACAGCGATA	This study
MEDG52_4928_1_coxL_1_f	GCTHGCAATAGCTATGGGTC	This study
MEDG52_4928_1_coxL_1_r	CCATAAGTGTACCRGTCTTC	This study
MEDG52_4929_1_coxL_1_f	TACGGCGCCTTATGGKYTRG	This study
MEDG52_4929_1_coxL_1_r	TCATAGTTTTGAAACGCTCR	This study
UBA1014_598_1_coxL_2_f	TTCCAACRGATACGGTGATG	This study
UBA1014_598_1_coxL_2_r	GCATCHGACTGCATAGACTT	This study
338f (bacterial 16S)	ACTCCTACGGGAGGCAGCAG	(65)
518r (bacterial 16S)	ATTACCGCGGCTGCTGG	(65)

^*a*^ Characters in front of the “coxL” in the names of primers correspond to the name of genera and species clusters of target bacteria.

Specific amplification was confirmed by gel electrophoresis and sequencing of the PCR products (Fig. S7). Copy density of *coxL* ranged from 2.47 × 10^3^ ± 3.27 × 10^2^ to 4.82 × 10^4^ ± 5.76 × 10^3^ cp/mL (1.03 × 10^5^ ± 7.63 × 10^3^ cp/mL in total) (Table 4), corresponding to 0.20%–3.96% (8.49% in total) of bacterial 16S rRNA genes (1.25 × 10^6^ ± 5.64 × 10^5^ cp/mL) (Table 4). Because most p*cox*-CO oxidizers contain a single *coxMSL* gene operon, whereas many bacteria encode multiple 16S rRNA copies (66), the proportion of p*cox*-CO oxidizers of these four species clusters likely exceeded 8.49%. Although the temporal dynamics and CO oxidation activity remain to be explored, our findings suggest an abundance of p*cox*-CO oxidizers in Osaka Bay at this time of year.

**TABLE 4:** Copy densities of *coxL* of analyzed species clusters and bacterial 16S rRNA^*a*^

Organism (phylum; genus; species cluster)	Target gene	Copy densities (cp/mL)	*R* ^2^	Eff
*Pseudomonadota*; LGRT01; 4908_1,4908_2,4908_3	*coxL*	2.4 × 10^4^ ± 4.3 ×10^3^	0.994	80.6
*Pseudomonadota*; MED-G52; 4928_1	*coxL*	2.9 × 10^4^ ± 2.6 × 10^3^	1.000	70.2
*Pseudomonadota*; MED-G52; 4929_1	*coxL*	4.8 × 10^4^ ± 5.8 × 10^3^	0.980	56.2
SAR324; UBA1014; 598_1	*coxL*	2.5 × 10^3^ ± 3.3 × 10^2^	0.997	87.3
Bacteria	16S rRNA	1.2 × 10^6^ ± 5.6 × 10^5^	0.989	99.3

^*a*^ Copy densities represent the average ± standard deviation obtained from triplicate analyses.

## DISCUSSION

In this study, we identified p*cox*-CO oxidizers from a large catalog of prokaryotic MAGs using stringent criteria that minimized overestimation. It resulted in a comprehensive and well-curated catalog comprising 233 species clusters, including 207 species clusters newly identified as p*cox*-CO oxidizers. Metagenome read mapping indicated that p*cox*-CO oxidizers in the catalog accounted for 0.08%–6.77% of prokaryotic communities across metagenomic clusters (i.e., oceanic regions) (Fig. 3A). These values were lower than previous estimates of 10%–20% (22, 32). Several factors may explain this discrepancy: (i) relatively low read recruitment, (ii) stringent criteria to identify p*cox*-CO oxidizers, which may exclude part of the true diversity, and (iii) diversification of the *coxMSL* gene operon among species-level populations, which may hinder read mapping to representative genomes. Despite potential underestimation from these factors, our findings suggest that p*cox*-CO oxidizers constitute at least several percent of the prokaryotic community across vast oceanic regions. This supports the notion that CO is an important energy source for marine prokaryotes (22, 23). Species-specific biogeographic analyses further revealed that 11 species clusters of p*cox*-CO oxidizers co-occurred with 20 species clusters of prokaryotes, providing clues about biotic factors influencing their distributions. Absolute quantification of four species clusters in Osaka Bay demonstrated substantial abundance of p*cox*-CO oxidizers.

Notably, co-occurring prokaryotes differed among species clusters of p*cox*-CO oxidizers (no more than two species clusters of p*cox*-CO oxidizers appeared within any co-occurrence module [Fig. 5]). Moreover, aside from genes related to CO oxidation, no shared genes were detected among p*cox*-CO oxidizers that co-occurred with other prokaryotes (Table 2). These findings suggest that Mo-CODH-mediated CO oxidation does not necessarily drive ecological interactions with surrounding prokaryotes. This notion contrasts with previous discussions on Ni‑CODH‑mediated CO oxidation, which propose that CO oxidation benefits surrounding prokaryotes by removing high concentrations of CO while supplying hydrogen as a metabolic product (67).

Overall, this study provides both a practical contribution for future ecological research on p*cox*-CO oxidizers and a theoretical contribution by offering a new insight into the ecological role of Mo-CODH-mediated CO oxidation.

## MATERIALS AND METHODS

### Investigating the predicted functional form I *coxL* and p*cox*-CO oxidizers

Given the known features of form I CoxL, we formulated three steps to identify form I CoxL: (i) phylogenetic relationships with reference CoxL, (ii) presence of active site motifs (17), and (iii) the *coxMSL* operon structure, in which *coxM*, *coxS*, and *coxL* occur in this order (17). To confirm the diversity of active site motifs, 707 CoxL (regarded as functional form I [23]) were aligned using L-INS-I method in MAFFT v.7.520 (68), and motifs were extracted using SeqKit v.2.8.2 (69).

To survey p*cox*-CO oxidizers, we prepared two data sets: the non-OceanDNA and OceanDNA MAGs. The non-OceanDNA MAGs included 107 isolate genomes and 1,221 MAGs/SAGs. The 107 isolates included five in which CO oxidation had been verified (21, 27), 98 that had *cox*, and 4 that belonged to the same subclade as *cox*-bearing prokaryotes (70). Among MAGs/SAGs, 640 encoded *cox* and 581 belonged to phyla or phylogenetic clades containing *cox*-bearing genomes (25, 30, 70–77). FASTA files of the non-OceanDNA MAGs were obtained from the National Center for Biotechnology Information (NCBI) RefSeq/GenBank (78, 79), figshare (80, 81), the European Molecular Biology Laboratory-European Bioinformatics Institute (82), and the China National Center for Bioinformation Genome Warehouse (83). FASTA files of the OceanDNA MAGs were retrieved from figshare (84). Genome completeness and contamination were re-analyzed using CheckM v.1.2.2 (85) for the non-OceanDNA MAGs and retrieved from literature for the OceanDNA MAGs (39). Protein-coding genes were predicted using Prodigal v.2.6.3 (86). CoxL-like proteins were identified using BLASTp of DIAMOND v.2.0.15 (87), using 707 reference CoxL (23) as queries (database size = 10^7^ bp, E-value ≤ 10^−5^). CoxL-like proteins with active site motifs were subjected to phylogenetic analysis using one form I CoxL (AEI08106.1) and three form II CoxL (WP_011083168.1, WP_010909926.1, WP_010970339.1) as references. The details of the phylogenetic analysis are provided in Supplemental methods. Proteins that formed a monophyletic group with AEI08106.1 were classified as form I CoxL. Genomes containing form I *coxL* were annotated using eggNOG-mapper v.2.1.11 (88). Five genes upstream of form I *coxL* were assessed, and loci encoding *coxM* (COG1319), *coxS* (COG2080), and form I *coxL* in this order were defined as *coxMSL* gene operons. We clustered the genomes with *coxMSL* gene operons at 95% ANI using dRep v.3.2.2 (89) to delineate species clusters. Phylogeny of representative genomes of species clusters was re-annotated using GTDB-Tk v.2.4.0 (58), and a phylogenetic tree of the representative genomes was constructed. The details of the analysis are provided in Supplemental methods.

### Mapping of the metagenome reads

Metagenome data of the 1,134 samples (Table S5) were downloaded from the NCBI Sequence Read Archive (SRA) (90) using SRA Toolkit v.3.0.5 (https://hpc.nih.gov/apps/sratoolkit.html), selecting run with the highest read count when multiple runs were performed. Environmental parameters were retrieved from the literature (84). Reads were pre-processed using Trimmomatic v.0.39 (91) to remove adapters and bases at either end of the reads that fell below a quality score of 20. Reads with a minimum length of 60 bp were retained. Mapping was performed against 8,466 representative genomes of species clusters of OceanDNA MAGs (39). If a representative genome of a species cluster containing p*cox*-CO oxidizers lacked *cox*, it was replaced with p*cox*-CO oxidizer MAG with the highest quality. The quality was calculated as follows: quality = completeness (%) – [5 × contamination (%)] (39).

Mapping was performed using Bowtie2 v.2.5.2 (92) with the default parameters. Reads aligned with identity ≥95%, mapped region ≥80 bp, and a fraction of mapped region on the reads ≥80% were retained using msamtools v.1.1.2 (https://github.com/arumugamlab/msamtools). Mapped reads were counted using Subread v.2.0.6 (https://subread.sourceforge.net/).

### Relative abundance of MAGs

Relative abundance of representative genomes was assessed to analyze prokaryotic community composition. First, we annotated genes in the representative genomes using Prodigal (86) and eggNOG-mapper v.2.1.11 (88) and retrieved the 14 universal single-copy marker genes (22) according to COG (Table S6). We counted reads mapped to each gene using Subread v.2.0.6 (https://subread.sourceforge.net/) and calculated RPKM values. The relative abundance of the representative genome of species cluster *i* was calculated using equation 1:


(1) $$(\text{relative abundance}{)}_{i}=\frac{x_{i}}{\sum_{k=1}^{n}x_{k}}$$

where *x_i_* represents the average RPKM value of the 14 marker genes in the representative genome of species cluster *i*.

To estimate the relative abundance of p*cox*-CO oxidizers, we counted reads mapped to *coxMSL* gene operons as stated above and calculated RPKM values. Relative abundance of representative genomes was calculated using equation 2:


(2) $$(\text{relative abundance}{)}_{i}=\frac{coxMSL_{i}}{\sum_{k=1}^{n}x_{k}}$$

where *coxMSL_i_* represents the RPKM value of the *coxMSL* gene operon of the representative genome of species cluster *i*, and *xk* represents the average RPKM value of the universal single-copy marker genes in the representative genome of species cluster *k*. The resultant values are given in Table S8.

### Statistical analyses

Statistical analyses were conducted in R (93) using vegan v.2.6.6.1 (94) package. We calculated Bray-Curtis dissimilarities between the 1,134 metagenome data sets based on the relative abundance of representative genomes of species clusters and clustered metagenome data using the hclust function in R. Distribution of sites where metagenome data were obtained was visualized using ggplot2 v.3.5.1 (https://ggplot2.tidyverse.org/). The optimal number of metagenomic clusters was determined using the R package ClusterStab v.1.76.0 (https://bioconductor.org/packages/release/bioc/html/clusterStab.html). In this analysis, Euclidean distance between all the pairs of metagenome data were calculated from prokaryotic community composition. One thousand replicates of randomly subsampled data sets (comprising 70% of metagenome data) were divided into 5–10 clusters. The similarity between resulting clusters was examined, and the number in which clusters resulting from different replicates that were the most similar was selected. The minimum cluster number of five was determined from the notion that prokaryotic communities differ between surface and deep ocean (52), across salinity (brackish water vs seawater) (53), and across latitude (polar vs temperate vs tropical) (54). The dissimilarity in the prokaryotic community was compared using non-metric multidimensional scaling on the Bray-Curtis distance matrix. The correlations between prokaryotic communities and environmental parameters were visualized using ggplot2 v.3.5.1 (https://ggplot2.tidyverse.org/).

Bray-Curtis dissimilarities between metagenome data were also calculated based on composition of p*cox*-CO oxidizers. A Mantel test was performed to assess correlations between composition of p*cox*-CO oxidizers, prokaryotic community composition, and environmental parameters (depth, latitude, longitude, temperature, salinity, and oxygen concentration). To characterize factors affecting abundance of p*cox*-CO oxidizers, we calculated Spearman’s correlations between all possible pairs of relative abundance of p*cox*-CO oxidizers in each species cluster, relative abundance of whole community of each species cluster, and environmental parameters using R package psych v.2.4.12 (https://cran.r-project.org/web/packages/psych/citation.html). Correlations with |*r*| values ≥ 0.85 and *P*-values <0.01 were retrieved as performed in a previous study (95). Correlations involving p*cox*-CO oxidizers of dominant species clusters but not involving the whole community of corresponding species clusters were retained. Co-occurrence networks were visualized using Cytoscape v.3.10.3 (96).

### Comparison of pangenome of p*cox*-CO oxidizers among species clusters

We retrieved *cox*-lacking MAGs in species clusters in which p*cox*-CO oxidizers were retained in the abovementioned co-occurrence network. The MAGs were annotated as stated above, and COGs present in any p*cox*-CO oxidizer MAG but absent from *cox*-lacking MAGs were identified.

### Collection and processing of seawater

Seawater for primer validation and quantification of p*cox*-CO oxidizers was collected from Osaka Bay, Japan (N 34°19′28′′, E 135°7′15′′) on 22 June, 20 July, and 16 November 2022. Detailed procedures for sampling, filtration, and DNA extraction are provided in Supplemental methods.

### Primer design and real-time PCR

We designed real-time primers specific to form I *coxL* of species clusters 4908_1, 4928_1, 4929_1, and 598_1. Copy density of *coxL* and bacterial 16S rRNA genes were quantified using primers listed in Table 3. Quality control for enumeration was achieved using dilution series of the PCR products. Additional details are provided in Supplemental methods.

## SUPPLEMENTAL MATERIAL

Supplemental material msphere.00062-26-s0001.docx Supplemental methods, results, figures, and references.

Supplemental tables msphere.00062-26-s0002.xlsx Tables S1 to S8.

ASM does not own the copyrights to Supplemental Material that may be linked to, or accessed through, an article. The authors have granted ASM a non-exclusive, world-wide license to publish the Supplemental Material files. Please contact the corresponding author directly for reuse.
