# Supplementary material for: Diversity and geographical distribution of potential carbon monoxide oxidizers using molybdenum-containing enzymes in the ocean
Source: mSphere. 2026 Apr 23;11(5):e00062-26. doi: 10.1128/msphere.00062-26 (PMC13203968; doi:10.1128/msphere.00062-26)
Supplement: Supplemental material — Supplemental methods, results, figures, and references. [file msphere.00062-26-s0001.docx]

**Supplementary methods**

Phylogenetic analysis of CoxL-like proteins.

CoxL-like proteins derived from the non-OceanDNA MAGs, the OceanDNA-MAGs, and the reference CoxL were aligned using L-INS-I method in MAFFT v.7.520 (1), trimmed using trimAl v.1.4.1 (2), which resulted in a dataset of 333 taxa and 779 sites from the non-OceanDNA MAGs and a dataset of 1,928 taxa and 726 sites from the OceanDNA MAGs. Maximum likelihood trees were obtained using IQTREE v. 2.2.2.6 (3) with 1,000 ultrafast bootstrap replicates. Based on the BIC, LG+F+I+R6 and LG+R7 substitution models were selected for the OceanDNA MAGs-derived and non-OceanDNA MAGs-derived datasets. The resultant trees were visualized using iTOL v. 6.8.2 (4).

Phylogenetic analysis of potential *cox*-containing carbon monoxide (CO) oxidizers (p*cox*-CO oxidizers) identified from the OceanDNA- and non-OceanDNA MAGs.

Multiple sequence alignments of conserved marker proteins encoded in metagenome assembled genomes (MAGs) of p*cox*-CO oxidizers were constructed using GTDB-Tk v. 2.4.0 (5), resulting in a dataset of 344 taxa and 5,035 sites. Maximum likelihood trees were obtained using IQTREE v. 2.2.2.6 (3) with 1,000 ultrafast bootstrap replicates. Based on the BIC, the LG+F+I+R10 substitution model was selected. The resultant trees were visualized using iTOL v. 6.8.2 (4).

Processing of seawater.

Five liters of seawater collected from 5 m depth of Osaka Bay, Japan (N 34°19′28′′, E 135°7′15′′) was filtered using a 3.0 µm pore size, hydrophilic polycarbonate membrane (Merck Millipore, Burlington, MA, USA) to remove eukaryotes. Prokaryotes were captured from three liters of flowthrough using STERIVEX filters (Merck Millipore) with 0.22 μm pore size by passing one liter of flowthrough through one filter. DNA was extracted using the DNeasy Power Water Kit (Qiagen, Hilden, Germany).

Primer design.

We designed real-time primers specific to form I *coxL* of the species-level genome clusters (species-clusters) 4908_1 (a member of the genus LGRT01, phylum *Pseudomonadota*), 4928_1 (a member of the uncultured genus MED-G52, phylum *Pseudomonadota*), 4929_1 (another member of the genus MED-G52), and 598_1 (an uncultured member of the genus UBA1014, phylum SAR324). We aligned *coxL* retrieved from genomes which belonged to the same genera to each species-cluster using MAFFT v.7.520 (1) and designed primers manually. Melting temperatures were calculated using Primer3Plus (6).

Assessment of primer specificity.

We evaluated the specificity of primer pairs on the National Center for Biotechnology Information (NCBI) nonredundant (nr) database as well as *coxL* found from the OceanDNA MAGs using Primer-BLAST (7). We performed an *in vitro* specificity test using environmental DNA from Osaka Bay as a template. The PCR mixture contained 0.125 μL of TaKaRa Ex Taq (TaKaRa Bio Inc., Kusatsu, Japan), 2.5 μL of 10×Ex Taq buffer (TaKaRa Bio Inc.), 2 μL of dNTP mixture (TaKaRa Bio Inc.), 0.5 μL each of 10 μM forward and reverse primer, 18.875 μL of virus-free water, and 0.5 μL of template. DNA extracted from seawater collected on June 22 was used to amplify *coxL* of 4908_1 and 4928_1, while DNA extracted from seawater collected on November 16 and July 20 was used to amplify *coxL* of 4929_1 and 598_1, respectively. PCR was run using TaKaRa PCR Thermal Cycler Dice Touch (TaKaRa Bio Inc.) under the following conditions: 1 min for initial denaturation at 95°C followed by 30 cycles of 5 s for denaturation at 95°C, 10 s for annealing at 58.4°C (to amplify *coxL* of 4908_1, 4928_1, and 4929_1) or 56.4°C (to amplify *coxL* of 598_1), and 30 s for extension at 72°C. The size of PCR products was confirmed using electrophoresis on 3% agarose gel. After purifying PCR products using the Wizard SV Gel and PCR Clean-Up System (Promega, Madison, WI, USA), the DNA concentrations were quantified using Qubit dsDNA Quantification Assay Kits (Thermo Fisher Scientific, Waltham, MA, USA). The copy densities of PCR products were calculated using following formula:

(Copy density (copies/μL)) = *D* × 6.022 × 10^23^/ (*L* × 660 × 10^9^)

where *D* represents the DNA concentration (ng/μL) and *L* represents the length (bp) of PCR products. Finally, PCR products were Sanger sequenced by Eurofins, Japan.

Polymerase chain reaction of 16S rRNA.

Genomic DNA was extracted from *E. coli* strain DH5α using DNeasy Blood & Tissue Kit (Qiagen) to prepare the standard for quantification of bacterial 16S rRNA. Universal real-time primers (338f/518r) (8) were used to amplify fragments of 16S rRNA. The composition of PCR mixture and conditions of PCR were the same as the amplification of *coxL*, except that annealing was performed at 61°C. PCR products were confirmed for size, purified, quantified, and sequenced as stated above.

Real-time PCR quantification.

We enumerated the copy density of *coxL* and bacterial 16S rRNA genes. A master mix was prepared for each well to contain the following: 12.5 μL of TB Green Premix Ex Taq II (TaKaRa Bio Inc.), forward and reverse primers at a concentration of 400 nM, and virus-free water to a total of 23 μL. Finally, 2.0 μL of sample or standard was added. Real time PCR was performed in triplicate using the Thermal Cycler Dice Real-Time System (TaKaRa Bio Inc.) under the following conditions: 30 s for initial denaturation at 95°C followed by 35–45 cycles of 5 s for denaturation at 94°C, 10 s for annealing at 55–63°C, and 20 s for extension at 72°C. Annealing was performed at 59.5°C, 58.4°C, 58.4°C, 55°C, and 63°C, and the cycle was repeated 35, 45, 45, 40, and 40 times for the quantification of *coxL* of 4908_1/4908_2/4908_3, *coxL* of 4928_1, *coxL* of 4929_1, *coxL* of 598_1, and bacterial 16S rRNA, respectively. A dissociation run was performed from 60 to 95°C. Standard curves were generated using a 10-fold dilution series of PCR products.

**Additional analyses and results**

Effect of inclusion of “AYRCSLR” motif on diversity and abundance of p*cox*-CO oxidizers.

To access whether the inclusion of “AYRCSLR” motif which lack the well-conserved “CSFR” residues (9) may lead to overestimation of the diversity and the abundance of p*cox*-CO oxidizers, we performed our screening process of p*cox*-CO oxidizers (Fig. S1) without this motif. As a result, the number of p*cox*-CO oxidizer genomes identified from the non-OceanDNA MAGs decreased from 235 to 232, while the number of p*cox*-CO oxidizer genomes identified from the OceanDNA MAGs was the same. The decrease of p*cox*-CO oxidizers resulted in decrease of species-clusters which were composed only of the non-OceanDNA MAGs (from 112 to 109), while the number of species-clusters which included the OceanDNA MAGs did not change. We thus concluded that the inclusion of the “AYRCSLR” motif did not lead to overestimation of diversity and abundance of p*cox*-CO oxidizers identified from the OceanDNA MAGs.

Characteristics of form I CoxL-like proteins with odd motifs.

When we retrieved CoxL-like proteins encoded in the OceanDNA MAGs, we identified two CoxL-like proteins from MAGs of one *Marinisomatota* and one *Pseudomonadota* bacterium (termed OceanDNA-b20244_00459_2 and OceanDNA-b26473_00006_19, respectively) which had active site-like motifs (AYRCSSR and AYRCSCR, respectively). To assess whether these CoxL-like proteins with odd motifs could function as bona fide form I Cox, we conducted phylogenetic analyses, examined the nucleotide sequences of their active sites, and investigated genomic context of genes encoding these proteins. Firstly, we constructed a maximum likelihood tree using the CoxL-like proteins with odd motifs, form I CoxL identified from the OceanDNA MAG, and three from II CoxL (WP_011083168.1, WP_010909926.1, and WP_010970339.1) as stated in the “Materials and methods” section. The resultant tree showed that the CoxL-like proteins with odd motifs were nested within form I CoxL clade and closely related to certain form I CoxL with conserved motifs with high non-parametric bootstrap supports >90% (Fig. S3). Secondly, we compared nucleic acid sequences encoding the odd motifs and those encoding genuine active site motifs of form I CoxL. By comparing the CoxL-like proteins with odd motifs with phylogenetically closely related form I CoxL (OceanDNA-b26467_00003_17 for OceanDNA-b26473_00006_19 and OceanDNA-b20250_00057_5 for OceanDNA-b20244_00459_2), we found the difference of active site motifs was attributed to substitutions of one nucleotide (Table S3). Thus, the odd motifs were not those of paralogs of CoxL with another enzymatic functions, but rather, most likely emerged very recently by point mutations from form I *coxL* with conserved motifs. Lastly, we performed functional annotation of genomes which encoded CoxL-like proteins with odd motifs (OceanDNA-b20244 and OceanDNA-b26473) using eggnog-mapper v. 2.1.11 and retrieved upstream of the genes encoding the CoxL-like proteins. We found that *coxM* (COG1319) and *coxS* (COG2080) were adjacent to gene encoding OceanDNA-b26473_00006_19 in this order, exhibiting the conserved gene order for functional form I CoxL. On the other hand, the genes adjacent to the gene encoding OceanDNA-b20244_00459_2 were not analyzed due to lack of the upstream region in short contig.

Therefore, it would be reasonable to include MAGs encompassing genes for CoxL-like proteins with odd motifs as candidates of potential CO oxidizer genomes. Nevertheless, the CoxL-like proteins with odd motifs might be non-functional or neofunctionalized, since activities of molybdenum-containing CO dehydrogenases with these motifs have not been reported.

**Supplementary figures**


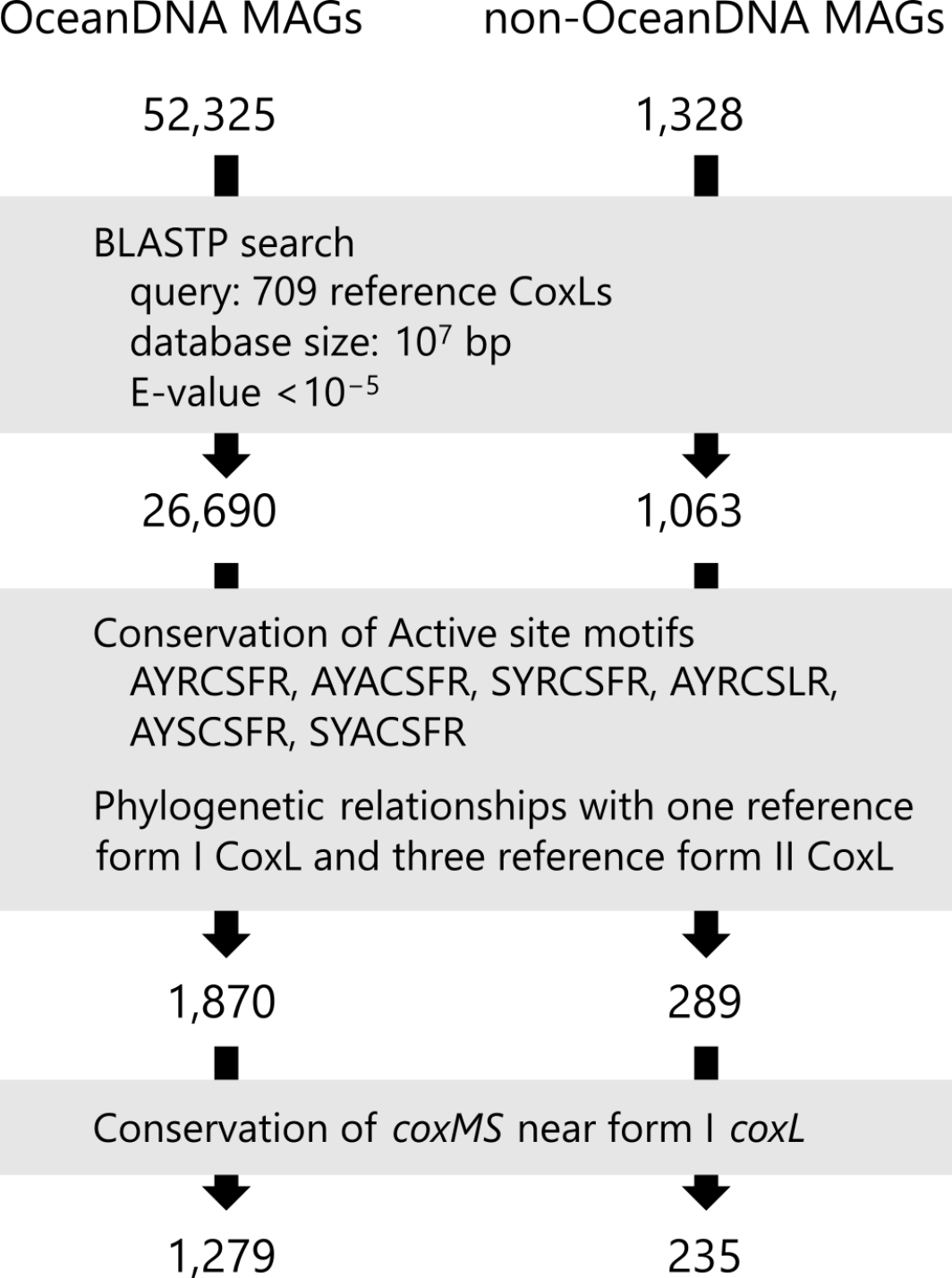


Fig. S1 Schematic representation of the pipeline to retrieve p*cox*-CO oxidizers. Numbers of the OceanDNA and non-OceanDNA MAGs retrieved in each process are represented on the left and right sides, respectively.


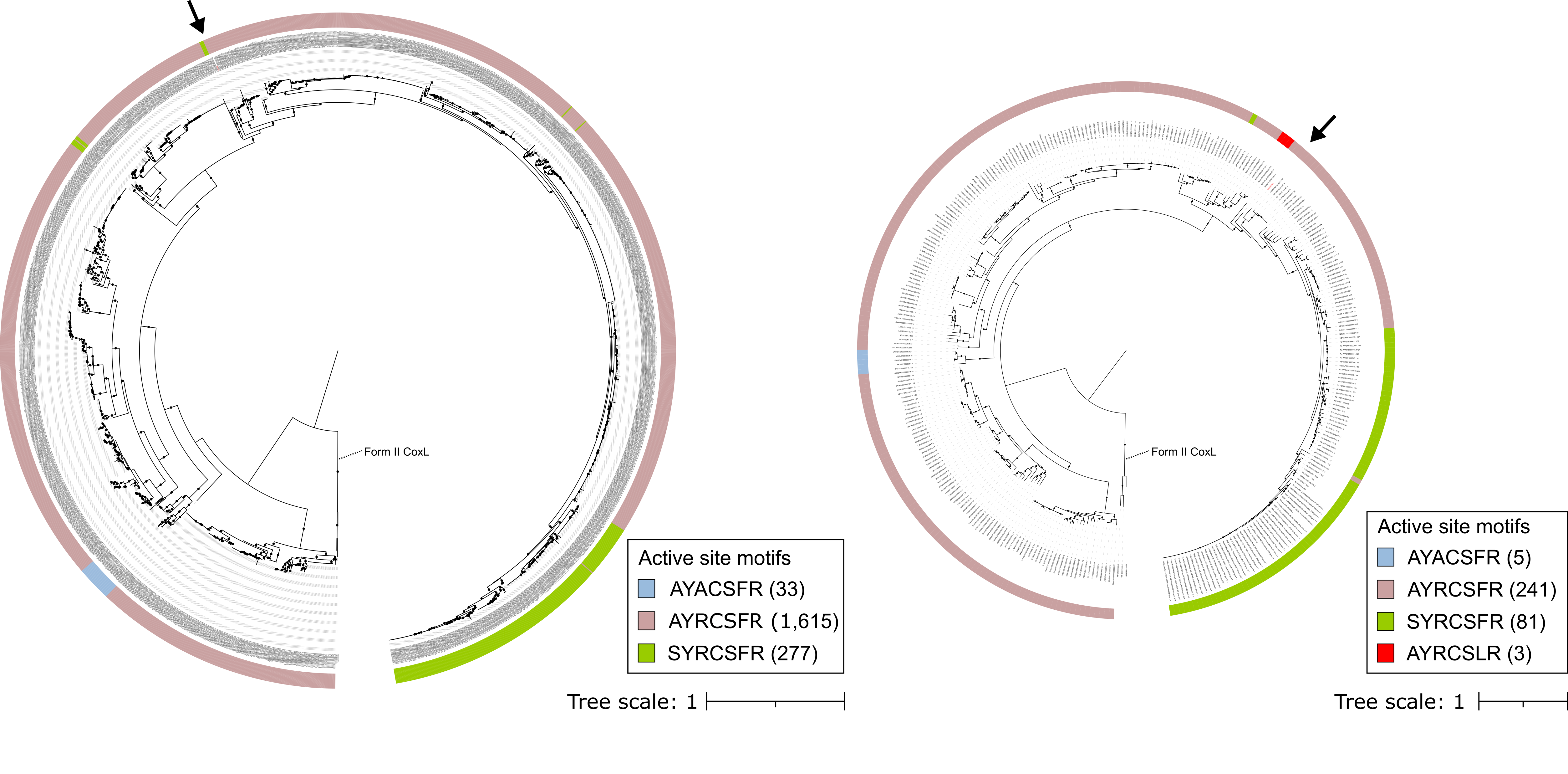


Fig. S2 Phylogenetic trees of CoxL-like proteins which conserved active site motifs of form I CoxL identified from the OceanDNA (left) and non-OceanDNA MAGs (right). Branches with bootstrap values ≥90 are highlighted by black dots. Branches of form II CoxL are indicated by dotted lines. Black arrows indicate the reference form I CoxL. Branch lengths represent the number of substitutions per site. The colors on the outer circle represent active site motifs. Numbers in the parenthesis indicates the numbers of form I CoxL with the corresponding active site motifs.


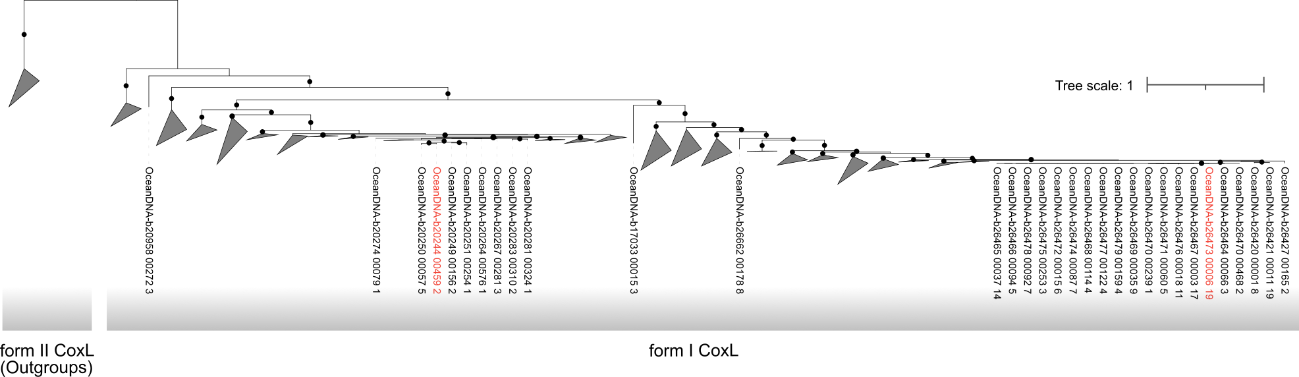


Fig. S3 Phylogenetic tree of the CoxL-like proteins with odd motifs, form I CoxL identified from the OceanDNA MAG, and three from II CoxL. The CoxL-like proteins with odd motifs and the form I CoxL are shown in red and black, respectively. Branches with bootstrap values of ≥90 are highlighted by black dots. Branch lengths represent the number of substitutions per site.


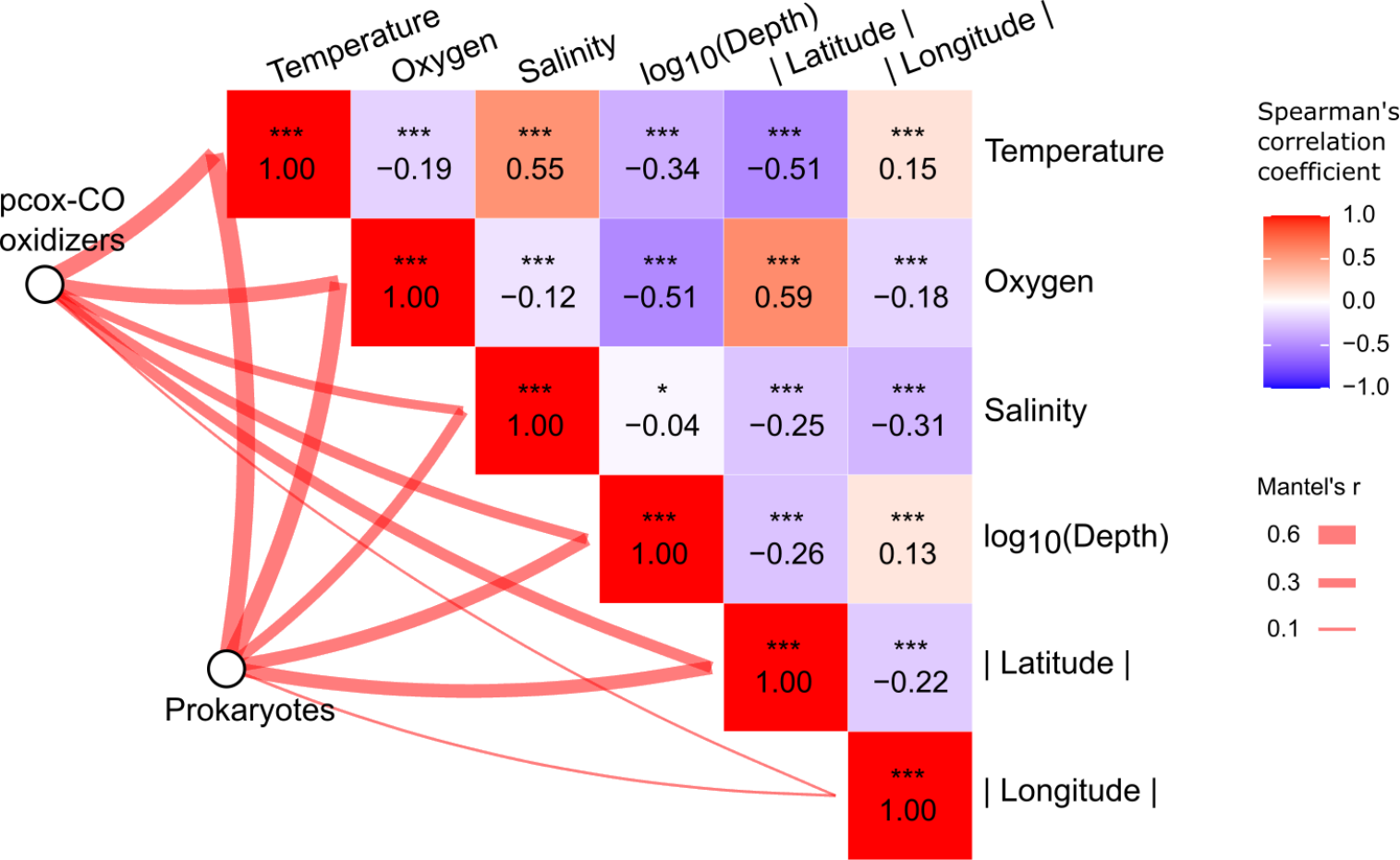


Fig. S4 Mantel tests and correlation matrix which show the relationships between environment parameters and community composition. Mantel tests show correlation between environmental parameters and community composition of p*cox*-CO oxidizers and between environmental parameters and prokaryotic community composition. The width of the red lines corresponds to the Mantel’s *r* statistics. Correlation matrix shows the Spearman’s correlation among environmental parameters. Red and blue represent the positive and negative correlations, respectively. Number in each box denotes the correlation coefficient value. Asterisks (*) denotes the significance levels: *** denotes *p*<0.001 and * denotes *p*>0.05.


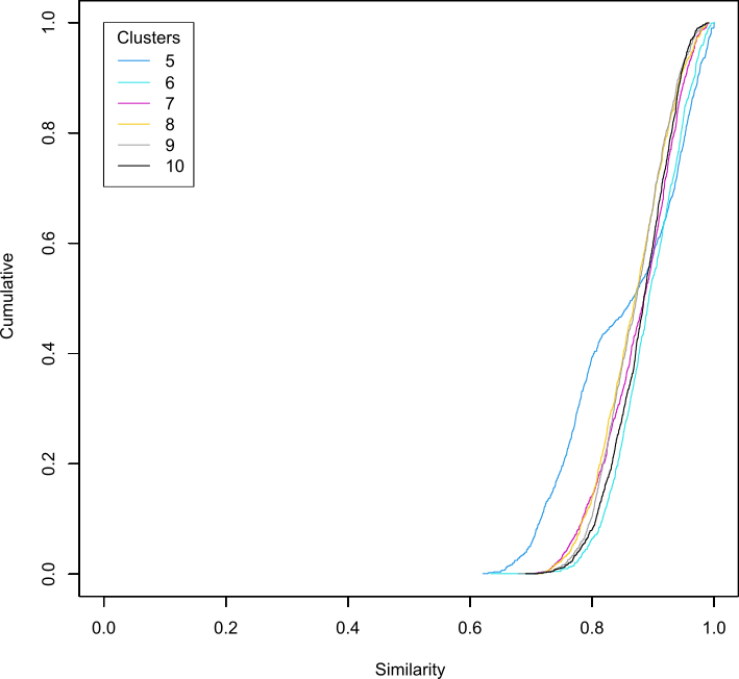


Fig. S5 Stability of the results of clustering of 1,134 metagenome datasets based on the community composition of the OceanDNA MAGs. Line plots show cumulative rate of comparison between different clustering attempts with each value of similarity.


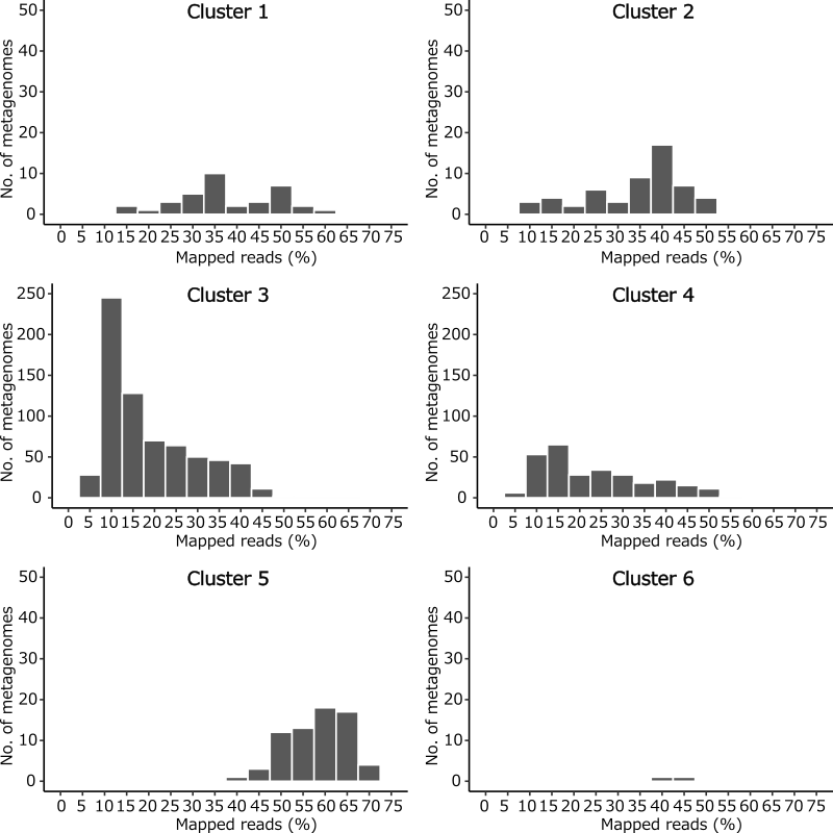


Fig. S6 Distributions of read recruitment of metagenome datasets which were assigned in each of six metagenomic cluster. The vertical axes represent the number of metagenome datasets, and the horizontal axes represent the read recruitment (proportion of the mapped reads to total reads).


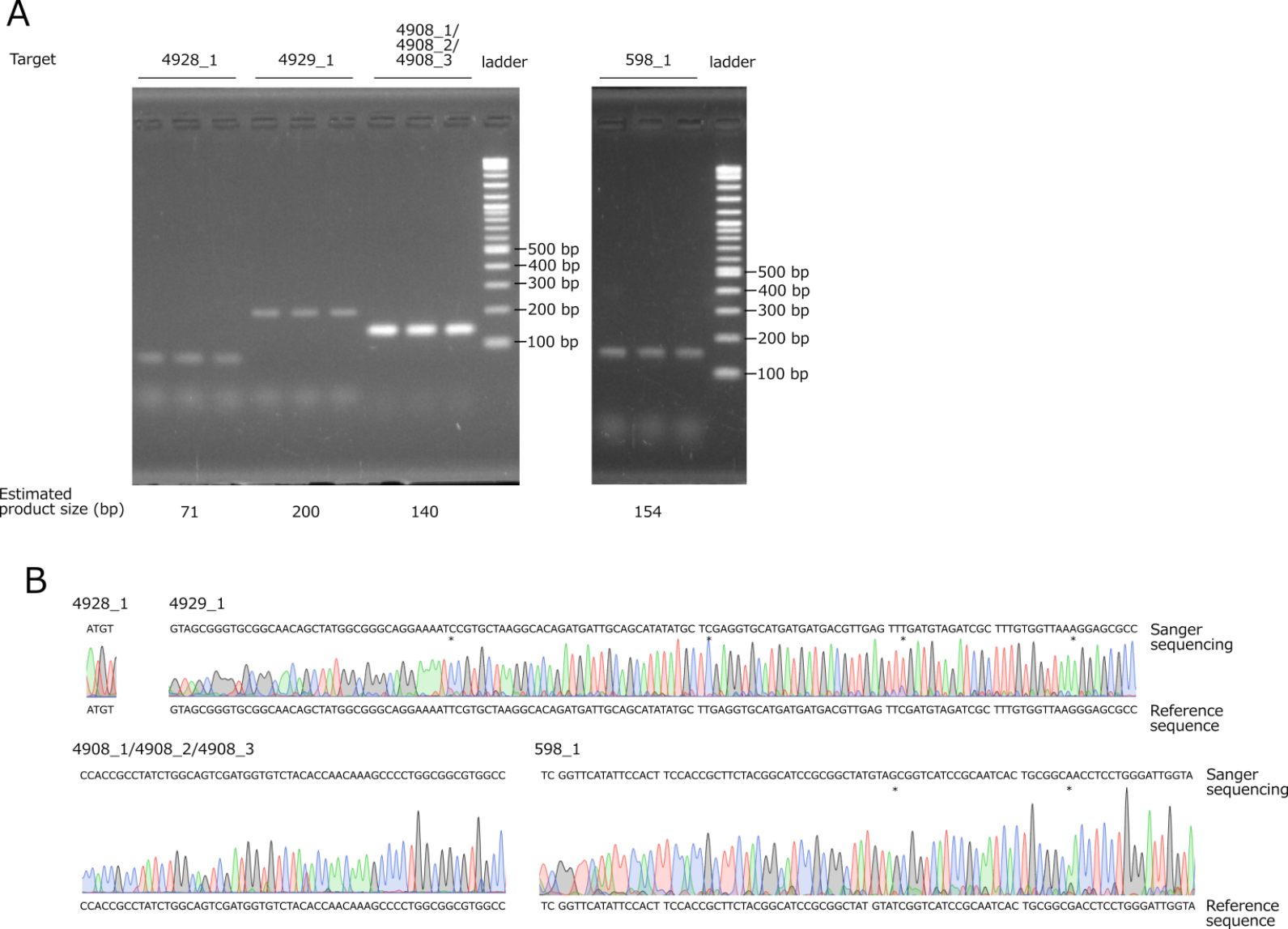


Fig. S7 Validation of real-time primers for species-specific absolute quantification of *coxL* genes. (A) Agarose gel image of PCR products amplified using real-time primers specific to *coxL* of p*cox*-CO oxidizers in the species-clusters 4928_1, 4929_1, 4908_1/4908_2/4908_3, and 598_1. (B) Sanger sequencing chromatogram of PCR product of *coxL* of each of the four species-cluster. Sequences resulting from the Sanger sequences and sequences of reference *coxL* are shown with chromatograms. Asterisks indicate bases that were different between the Sanger sequencing-derived and reference *coxL* sequences.

**References for supplementary methods**

1. Katoh K, Toh H. 2008. Recent developments in the MAFFT multiple sequence alignment program. Brief Bioinform 9:286–298.

2. Capella-Gutiérrez S, Silla-Martínez JM, Gabaldón T. 2009. trimAl: a tool for automated alignment trimming in large-scale phylogenetic analyses. Bioinformatics 25:1972–1973.

3. Minh BQ, Schmidt HA, Chernomor O, Schrempf D, Woodhams MD, von Haeseler A, Lanfear R. 2020. IQ-TREE 2: new models and efficient methods for phylogenetic inference in the genomic era. Mol Biol Evol 37:1530–1534.

4. Letunic I, Bork P. 2021. Interactive Tree Of Life (iTOL) v5: an online tool for phylogenetic tree display and annotation. Nucleic Acids Res 49:W293–W296.

5. Chaumeil PA, Mussig AJ, Hugenholtz P, Parks DH. 2022. GTDB-Tk v2: memory friendly classification with the genome taxonomy database. Bioinformatics 38:5315–5316.

6. Untergasser A, Nijveen H, Rao X, Bisseling T, Geurts R, Leunissen JAM. 2007. Primer3Plus, an enhanced web interface to Primer3. Nucleic Acids Res 35:W71–W74.

7. Ye J, Coulouris G, Zaretskaya I, Cutcutache I, Rozen S, Madden TL. 2012. Primer-BLAST: a tool to design target-specific primers for polymerase chain reaction. BMC Bioinformatics 13:134.

8. Einen J, Thorseth IH, Øvreås L. 2008. Enumeration of Archaea and Bacteria in seafloor basalt using real-time quantitative PCR and fluorescence microscopy. FEMS Microbiol Lett 282:182–187.

9. Dunfield KE, King GM. 2004. Molecular analysis of carbon monoxide-oxidizing bacteria associated with recent Hawaiian volcanic deposits. Appl Environ Microbiol 70:4242–4248.

**References for supplementary tables**

1. Kim BC, Park JR, Bae JW, Rhee SK, Kim KH, Oh JW, Park YH. 2006. *Stappia marina* sp. nov., a marine bacterium isolated from the Yellow Sea. Int J Syst Evol Microbiol 56:75–79.

2. Bauer M, Kube M, Teeling H, Richter M, Lombardot T, Allers E, Würdemann CA, Quast C, Kuhl H, Knaust F, Woebken D, Bischof K, Mussmann M, Choudhuri JV, Meyer F, Reinhardt R, Amann RI, Glöckner FO. 2006. Whole genome analysis of the marine Bacteroidetes*‘Gramella forsetii’* reveals adaptations to degradation of polymeric organic matter. Environ Microbiol 8:2201–2213.

3. Dick GJ, Podell S, Johnson HA, Rivera-Espinoza Y, Bernier-Latmani R, McCarthy JK, Torpey JW, Clement BG, Gaasterland T, Tebo BM. 2008. Genomic insights into Mn(II) oxidation by the marine Alphaproteobacterium *Aurantimonas* sp. strain SI85-9A1. Appl Environ Microbiol 74:2646–2658.

4. Kang I, Oh HM, Lim SI, Ferriera S, Giovannoni SJ, Cho JC. 2010. Genome sequence of *Fulvimarina pelagi* HTCC2506^T^, a Mn(II)-oxidizing Alphaproteobacterium possessing an aerobic anoxygenic photosynthetic gene cluster and xanthorhodopsin. J Bacteriol 192:4798–4799.

5. Cunliffe M. 2011. Correlating carbon monoxide oxidation with *cox* genes in the abundant Marine *Roseobacter* Clade. ISME J 5:685–691.

6. Grote J, Bayindirli C, Bergauer K, Carpintero de Moraes P, Chen H, D’Ambrosio L, Edwards B, Fernández Gómez B, Hamisi M, Logares R, Nguyen D, Rii YM, Saeck E, Schutte C, Widner B, Church MJ, Steward GF, Karl DM, DeLong EF, Eppley JM, Schuster SC, Kyrpides NC, Rappé MS. 2011. Draft genome sequence of strain HIMB100, a cultured representative of the SAR116 clade of marine *Alphaproteobacteria*. Stand Genomic Sci 5:269–278.

7. Grote J, Thrash JC, Huggett MJ, Landry ZC, Carini P, Giovannoni SJ, Rappé MS. 2012. Streamlining and core genome conservation among highly divergent members of the SAR11 clade. mBio 3:e00252-12.

8. Swan BK, Tupper B, Sczyrba A, Lauro FM, Martinez-Garcia M, González JM, Luo H, Wright JJ, Landry ZC, Hanson NW, Thompson BP, Poulton NJ, Schwientek P, Acinas SG, Giovannoni SJ, Moran MA, Hallam SJ, Cavicchioli R, Woyke T, Stepanauskas R. 2013. Prevalent genome streamlining and latitudinal divergence of planktonic bacteria in the surface ocean. Proc Natl Acad Sci U S A 110:11463–11468.

9. Bondarev V, Richter M, Romano S, Piel J, Schwedt A, Schulz-Vogt HN. 2013. The genus *Pseudovibrio* contains metabolically versatile bacteria adapted for symbiosis. Environ Microbiol 15:2095–2113.

10. Voget S, Wemheuer B, Brinkhoff T, Vollmers J, Dietrich S, Giebel HA, Beardsley C, Sardemann C, Bakenhus I, Billerbeck S, Daniel R, Simon M. 2015. Adaptation of an abundant *Roseobacter* RCA organism to pelagic systems revealed by genomic and transcriptomic analyses. ISME J 9:371–384.

11. Riedel T, Fiebig A, Göker M, Klenk HP. 2014. Complete genome sequence of the bacteriochlorophyll a-containing *Roseibacterium elongatum* type strain (DSM 19469^T^), a representative of the *Roseobacter* group isolated from Australian coast sand. Stand Genomic Sci 9:840–854.

12. Durham BP, Grote J, Whittaker KA, Bender SJ, Luo H, Grim SL, Brown JM, Casey JR, Dron A, Florez-Leiva L, Krupke A, Luria CM, Mine AH, Nigro OD, Pather S, Talarmin A, Wear EK, Weber TS, Wilson JM, Church MJ, DeLong EF, Karl DM, Steward GF, Eppley JM, Kyrpides NC, Schuster S, Rappé MS. 2014. Draft genome sequence of marine alphaproteobacterial strain HIMB11, the first cultivated representative of a unique lineage within the *Roseobacter* clade possessing an unusually small genome. Stand Genomic Sci 9:632–645.

13. Mas-Lladó M, Piña-Villalonga JM, Brunet-Galmés I, Nogales B, Bosch R. 2014. Draft genome sequences of two isolates of the *Roseobacter* group, *Sulfitobacter* sp. strains 3SOLIMAR09 and 1FIGIMAR09, from harbors of Mallorca Island (Mediterranean Sea). Genome Announc 2:e00350-14.

14. Luo H, Moran MA. 2014. Evolutionary ecology of the Marine *Roseobacter* Clade. Microbiol Mol Biol Rev 78:573–587.

15. Mizuno CM, Rodriguez-Valera F, Ghai R. 2015. Genomes of planktonic *Acidimicrobiales*: widening horizons for marine Actinobacteria by metagenomics. mBio 6:e02083-14.

16. Billerbeck S, Wemheuer B, Voget S, Poehlein A, Giebel HA, Brinkhoff T, Gram L, Jeffrey WH, Daniel R, Simon M. 2016. Biogeography and environmental genomics of the *Roseobacter*-affiliated pelagic CHAB-I-5 lineage. Nat Microbiol 1:1–8.

17. Giebel HA, Klotz F, Voget S, Poehlein A, Grosser K, Teske A, Brinkhoff T. 2016. Draft genome sequence of the marine *Rhodobacteraceae* strain O3.65, cultivated from oil-polluted seawater of the Deepwater Horizon oil spill. Stand Genomic Sci 11:81.

18. Rodrigo-Torres L, Pujalte MJ, Arahal DR. 2016. Draft genomes of *Nautella italica* strains CECT 7645^T^ and CECT 7321: two roseobacters with potential pathogenic and biotechnological traits. Mar Genom 26:73–80.

19. Zhang Y, Sun Y, Jiao N, Stepanauskas R, Luo H. 2016. Ecological genomics of the uncultivated marine *Roseobacter* lineage CHAB-I-5. Appl Environ Microbiol 82:2100–2111.

20. Mehrshad M, Amoozegar MA, Ghai R, Shahzadeh Fazeli SA, Rodriguez-Valera F. 2016. Genome reconstruction from metagenomic data sets reveals novel microbes in the brackish waters of the Caspian Sea. Appl Environ Microbiol 82:1599–1612.

21. Bertagnolli AD, Padilla CC, Glass JB, Thamdrup B, Stewart FJ. 2017. Metabolic potential and in situ activity of marine *Marinimicrobia* bacteria in an anoxic water column. Environ Microbiol 19:4392–4416.

22. Hou L, Sun J, Xie X, Jiao N, Zhang Y. 2017. Genome sequence of *Acuticoccus yangtzensis* JL1095^T^ (DSM 28604^T^) isolated from the Yangtze Estuary. Stand Genomic Sci 12:91.

23. Landry Z, Swan BK, Herndl GJ, Stepanauskas R, Giovannoni SJ. 2017. SAR202 genomes from the dark ocean predict pathways for the oxidation of recalcitrant dissolved organic matter. mBio 8:e00413-17.

24. Pujalte MJ, Lucena T, Rodrigo-Torres L, Arahal DR. 2018. Comparative genomics of *Thalassobius* including the description of *Thalassobius activus* sp. nov., and *Thalassobius autumnalis* sp. nov. Front Microbiol 8:2645.

25. Haro-Moreno JM, Rodriguez-Valera F, Rosselli R, Martinez-Hernandez F, Roda-Garcia JJ, Gomez ML, Fornas O, Martinez-Garcia M, López-Pérez M. 2020. Ecogenomics of the SAR11 clade. Environ Microbiol 22:1748–1763.

26. Zheng Q, Lu J, Wang Y, Jiao N. 2019. Genomic reconstructions and potential metabolic strategies of generalist and specialist heterotrophic bacteria associated with an estuary *Synechococcus* culture. FEMS Microbiol Ecol 95:fiz017.

27. Gao ZM, Huang JM, Cui GJ, Li WL, Li J, Wei ZF, Chen J, Xin YZ, Cai DS, Zhang AQ, Wang Y. 2019. In situ meta-omic insights into the community compositions and ecological roles of hadal microbes in the Mariana Trench. Environ Microbiol 21:4092–4108.

28. Lee J, Kwon KK, Lim SI, Song J, Choi AR, Yang SH, Jung KH, Lee JH, Kang SG, Oh HM, Cho JC. 2019. Isolation, cultivation, and genome analysis of proteorhodopsin-containing SAR116-clade strain *Candidatus* *Puniceispirillum* *marinum* IMCC1322. J Microbiol 57:676–687.

29. Rambo IM, Dombrowski N, Constant L, Erdner D, Baker BJ. 2020. Metabolic relationships of uncultured bacteria associated with the microalgae *Gambierdiscus*. Environ Microbiol 22:1764–1783.

30. Cui G, Zhou Y, Li W, Gao Z, Huang J, Wang Y. 2021. A novel bacterial phylum that participates in carbon and osmolyte cycling in the Challenger Deep sediments. Environ Microbiol 23:3758–3772.

31. Liu J, Xue CX, Sun H, Zheng Y, Meng Z, Zhang XH. 2019. Carbohydrate catabolic capability of a *Flavobacteriia* bacterium isolated from hadal water. Syst Appl Microbiol 42:263–274.

32. Hollensteiner J, Schneider D, Poehlein A, Daniel R. 2020. Complete genome of *Roseobacter ponti* DSM 106830^T^. Genome Biol Evol 12:1013–1018.

33. Zhou Z, Tran PQ, Kieft K, Anantharaman K. 2020. Genome diversification in globally distributed novel marine *Proteobacteria* is linked to environmental adaptation. ISME J 14:2060–2077.

34. Lu R, Gao ZM, Li WL, Wei ZF, Wei TS, Huang JM, Li M, Tao J, Wang HB, Wang Y. 2021. Asgard archaea in the haima cold seep: spatial distribution and genomic insights. Deep Sea Res 1 Oceanogr Res Pap 170:103489.

35. Zhong H, Sun H, Liu R, Zhan Y, Huang X, Ju F, Zhang XH. 2021. Comparative genomic analysis of *Labrenzia aggregata* (*Alphaproteobacteria*) strains isolated from the Mariana Trench: insights into the metabolic potentials and biogeochemical functions. Front Microbiol 12:770370.

36. Royo-Llonch M, Sánchez P, Ruiz-González C, Salazar G, Pedrós-Alió C, Sebastián M, Labadie K, Paoli L, M. Ibarbalz F, Zinger L, Churcheward B, Chaffron S, Eveillard D, Karsenti E, Sunagawa S, Wincker P, Karp-Boss L, Bowler C, Acinas SG. 2021. Compendium of 530 metagenome-assembled bacterial and archaeal genomes from the polar Arctic Ocean. Nat Microbiol 6:1561–1574.

37. Ruiz-Perez CA, Bertagnolli AD, Tsementzi D, Woyke T, Stewart FJ, Konstantinidis KT. 2021. Description of *Candidatus* *Mesopelagibacter* *carboxydoxydans* and *Candidatus* *Anoxipelagibacter* *denitrificans*: nitrate-reducing SAR11 genera that dominate mesopelagic and anoxic marine zones. Syst Appl Microbiol 44:126185.

38. Savoie ER, Lanclos VC, Henson MW, Cheng C, Getz EW, Barnes SJ, LaRowe DE, Rappé MS, Thrash JC. 2021. Ecophysiology of the cosmopolitan OM252 bacterioplankton (*Gammaproteobacteria*). mSystems 6:e0027621.

39. Langwig MV, De Anda V, Dombrowski N, Seitz KW, Rambo IM, Greening C, Teske AP, Baker BJ. 2022. Large-scale protein level comparison of *Deltaproteobacteria* reveals cohesive metabolic groups. ISME J 16:307–320.

40. Feng X, Chu X, Qian Y, Henson MW, Lanclos VC, Qin F, Zhao Y, Thrash JC, Luo H. 2021. Mechanisms driving genome reduction of a novel *Roseobacter* lineage showing vitamin B_12_ auxotrophy. bioRxiv https://doi.org/10.1101/2021.01.15.426902.

41. Chen YJ, Leung PM, Wood JL, Bay SK, Hugenholtz P, Kessler AJ, Shelley G, Waite DW, Franks AE, Cook PLM, Greening C. 2021. Metabolic flexibility allows bacterial habitat generalists to become dominant in a frequently disturbed ecosystem. ISME J 15:2986–3004.

42. Boeuf D, Eppley JM, Mende DR, Malmstrom RR, Woyke T, DeLong EF. 2021. Metapangenomics reveals depth-dependent shifts in metabolic potential for the ubiquitous marine bacterial SAR324 lineage. Microbiome 9:172.

43. Sun Y, Debeljak P, Obernosterer I. 2021. Microbial iron and carbon metabolism as revealed by taxonomy-specific functional diversity in the Southern Ocean. ISME J 15:2933–2946.

44. Luo D, Wang X, Feng X, Tian M, Wang S, Tang SL, Ang P, Yan A, Luo H. 2021. Population differentiation of *Rhodobacteraceae* along with coral compartments. ISME J 15:3286–3302.

45. Wei Z, Li Q, Lu R, Zheng P, Wang Y. 2022. In situ genomics and transcriptomics of SAR202 subclusters revealed subtle distinct activities in deep-sea water. Microorganisms 10:1629.

46. Burgsdorf I, Sizikov S, Squatrito V, Britstein M, Slaby BM, Cerrano C, Handley KM, Steindler L. 2022. Lineage-specific energy and carbon metabolism of sponge symbionts and contributions to the host carbon pool. ISME J 16:1163–1175.

47. Malfertheiner L, Martínez-Pérez C, Zhao Z, Herndl GJ, Baltar F. 2022. Phylogeny and metabolic potential of the candidate phylum SAR324. Biology 11:599.

48. Mayali X, Samo T, Kimbrel J, Stuart RK, Morris M, Rolison K, Ramon C, Kim YM, Munoz-Munoz N, Nicora C, Purvine S, Lipton M, Weber PK. 2022. Single cell carbon and nitrogen incorporation and remineralization profiles are uncoupled from phylogenetic groupings of diatom-associated bacteria. bioRxiv https://doi.org/10.1101/2022.07.01.498368.

49. Davenport EJ, Bose A. 2022. Taxonomic re-evaluation and genomic comparison of novel extracellular electron uptake-capable *Rhodovulum visakhapatnamense* and *Rhodovulum sulfidophilum* isolates. Microorganisms 10:1235.

50. Priest T, von Appen WJ, Oldenburg E, Popa O, Torres-Valdés S, Bienhold C, Metfies K, Boulton W, Mock T, Fuchs BM, Amann R, Boetius A, Wietz M. 2023. Atlantic water influx and sea-ice cover drive taxonomic and functional shifts in Arctic marine bacterial communities. ISME J 17:1612–1625.

51. Nguyen VH, Wemheuer B, Song W, Bennett H, Palladino G, Burgsdorf I, Sizikov S, Steindler L, Webster NS, Thomas T. 2023. Functional characterization and taxonomic classification of novel gammaproteobacterial diversity in sponges. Syst Appl Microbiol 46:126401.

52. Hameed A, Suchithra KV, Lin SY, Stothard P, Young CC. 2023. Genomic potential for inorganic carbon sequestration and xenobiotic degradation in marine bacterium *Youngimonas vesicularis* CC-AMW-E^T^ affiliated to family *Paracoccaceae*. Antonie Van Leeuwenhoek 116:1247–1259.

53. Nguyen VH, Wemheuer B, Song W, Bennett H, Webster N, Thomas T. 2023. Identification, classification, and functional characterization of novel sponge-associated acidimicrobiial species. Syst Appl Microbiol 46:126426.

54. Liu Y, Brinkhoff T, Berger M, Poehlein A, Voget S, Paoli L, Sunagawa S, Amann R, Simon M. 2023. Metagenome-assembled genomes reveal greatly expanded taxonomic and functional diversification of the abundant marine *Roseobacter* RCA cluster. Microbiome 11:265.

55. Dong X, Lan H, Huang L, Zhang H, Lin X, Weng S, Peng Y, Lin J, Wang J, Peng J, Yang Y. 2023. Metagenomic views of microbial communities in sand sediments associated with coral reefs. Microb Ecol 85:465–477.

56. Lappan R, Shelley G, Islam ZF, Leung PM, Lockwood S, Nauer PA, Jirapanjawat T, Ni G, Chen YJ, Kessler AJ, Williams TJ, Cavicchioli R, Baltar F, Cook PLM, Morales SE, Greening C. 2023. Molecular hydrogen in seawater supports growth of diverse marine bacteria. Nat Microbiol 8:581–595.

57. Mujakić I, Cabello-Yeves PJ, Villena-Alemany C, Piwosz K, Rodriguez-Valera F, Picazo A, Camacho A, Koblížek M. 2023. Multi-environment ecogenomics analysis of the cosmopolitan phylum *Gemmatimonadota*. Microbiol Spectr 11:e01112-23.

58. Huang J, Wang J, Li G, Lai Q, Zhu X, Wang S, Liu X, Shao Z, Wang L. 2023. *Pseudodonghicola flavimaris* sp. nov. and *Sedimentitalea xiamensis* sp. nov., two novel species belonging to the family *Roseobacteraceae*. Int J Syst Evol Microbiol 73:006192.

59. Zhu X, Wang L, Lai Q, Wang J, Huang J, Li G, Zeng L, Xia J, Shao Z. 2023. *Pseudophaeobacter profundi* sp. nov., isolated from the Western Pacific Ocean. Int J Syst Evol Microbiol 73:006071.

60. Nishimura Y, Yoshizawa S. 2022. The OceanDNA MAG catalog contains over 50,000 prokaryotic genomes originated from various marine environments. Sci Data 9:305.

61. Alneberg J, Sundh J, Bennke C, Beier S, Lundin D, Hugerth LW, Pinhassi J, Kisand V, Riemann L, Jürgens K, Labrenz M, Andersson AF. 2018. BARM and BalticMicrobeDB, a reference metagenome and interface to meta-omic data for the Baltic Sea. Sci Data 5:180146.

62. Galand PE, Pereira O, Hochart C, Jean CA, Didier D. 2018. A strong link between marine microbial community composition and function challenges the idea of functional redundancy. ISME J 12:2470–2478.

63. Sunagawa S, Coelho LP, Chaffron S, Kultima JR, Labadie K, Salazar G, Djahanschiri B, Zeller G, Mende DR, Alberti A, Cornejo-Castillo FM, Costea PI, Cruaud C, d’Ovidio F, Engelen S, Ferrera I, Gasol JM, Guidi L, Hildebrand F, Kokoszka F, Lepoivre C, Lima-Mendez G, Poulain J, Poulos BT, Royo-Llonch M, Sarmento H, Vieira-Silva S, Dimier C, Picheral M, Searson S, Kandels-Lewis S, Tara Oceans coordinators, Bowler C, de Vargas C, Gorsky G, Grimsley N, Hingamp P, Iudicone D, Jaillon O, Not F, Ogata H, Pesant S, Speich S, Stemmann L, Sullivan MB, Weissenbach J, Wincker P, Karsenti E, Raes J, Acinas SG, Bork P. 2015. Structure and function of the global ocean microbiome. Science 348:1261359.

64. Tara Oceans Consortium C, Tara Oceans Expedition P. 2017. Registry of all samples from the Tara Oceans Expedition (2009-2013). dataset publication series. PANGAEA. https://doi.pangaea.de/10.1594/PANGAEA.875582.

65. Tsementzi D, Wu J, Deutsch S, Nath S, Rodriguez-R LM, Burns AS, Ranjan P, Sarode N, Malmstrom RR, Padilla CC, Stone BK, Bristow LA, Larsen M, Glass JB, Thamdrup B, Woyke T, Konstantinidis KT, Stewart FJ. 2016. SAR11 bacteria linked to ocean anoxia and nitrogen loss. Nature 536:179–183.

66. Haroon MF, Thompson LR, Parks DH, Hugenholtz P, Stingl U. 2016. A catalogue of 136 microbial draft genomes from Red Sea metagenomes. Sci Data 3:160050.

67. Hawley AK, Torres-Beltrán M, Zaikova E, Walsh DA, Mueller A, Scofield M, Kheirandish S, Payne C, Pakhomova L, Bhatia M, Shevchuk O, Gies EA, Fairley D, Malfatti SA, Norbeck AD, Brewer HM, Pasa-Tolic L, del Rio TG, Suttle CA, Tringe S, Hallam SJ. 2017. A compendium of multi-omic sequence information from the Saanich Inlet water column. Sci Data 4:170160.

68. Acinas SG, Sánchez P, Salazar G, Cornejo-Castillo FM, Sebastián M, Logares R, Royo-Llonch M, Paoli L, Sunagawa S, Hingamp P, Ogata H, Lima-Mendez G, Roux S, González JM, Arrieta JM, Alam IS, Kamau A, Bowler C, Raes J, Pesant S, Bork P, Agustí S, Gojobori T, Vaqué D, Sullivan MB, Pedrós-Alió C, Massana R, Duarte CM, Gasol JM. 2021. Deep ocean metagenomes provide insight into the metabolic architecture of bathypelagic microbial communities. Commun Biol 4:1–15.

69. Bergauer K, Fernandez-Guerra A, Garcia JAL, Sprenger RR, Stepanauskas R, Pachiadaki MG, Jensen ON, Herndl GJ. 2018. Organic matter processing by microbial communities throughout the Atlantic water column as revealed by metaproteomics. Proc Natl Acad Sci U S A 115:E400–E408.

70. Mende DR, Bryant JA, Aylward FO, Eppley JM, Nielsen T, Karl DM, DeLong EF. 2017. Environmental drivers of a microbial genomic transition zone in the ocean’s interior. Nat Microbiol 2:1367–1373.

71. C-MORE : HOE-Legacy. https://hahana.soest.hawaii.edu/hoelegacy/hoelegacy.html.

72. Biller SJ, Berube PM, Dooley K, Williams M, Satinsky BM, Hackl T, Hogle SL, Coe A, Bergauer K, Bouman HA, Browning TJ, De Corte D, Hassler C, Hulston D, Jacquot JE, Maas EW, Reinthaler T, Sintes E, Yokokawa T, Chisholm SW. 2018. Marine microbial metagenomes sampled across space and time. Sci Data 5:180176.

73. Nowinski B, Smith CB, Thomas CM, Esson K, Marin R, Preston CM, Birch JM, Scholin CA, Huntemann M, Clum A, Foster B, Foster B, Roux S, Palaniappan K, Varghese N, Mukherjee S, Reddy TBK, Daum C, Copeland A, Chen IMA, Ivanova NN, Kyrpides NC, Glavina del Rio T, Whitman WB, Kiene RP, Eloe-Fadrosh EA, Moran MA. 2019. Microbial metagenomes and metatranscriptomes during a coastal phytoplankton bloom. Sci Data 6:129.
